# Polyhydroxyalkanoates: Medical Applications and Potential for Use in Dentistry

**DOI:** 10.3390/ma17225415

**Published:** 2024-11-06

**Authors:** Rim Ben Abdeladhim, José Alexandre Reis, Ana Maria Vieira, Catarina Dias de Almeida

**Affiliations:** 1 Egas Moniz School of Health & Science, Campus Universitário, Quinta da Granja, 2829-511 Caparica, Portugal; 116535@alunos.egasmoniz.edu.pt (R.B.A.); jreis@egasmoniz.edu.pt (J.A.R.); asvieira@egasmoniz.edu.pt (A.M.V.); 2 Egas Moniz Center for Interdisciplinary Research (CiiEM), Egas Moniz School of Health & Science, Campus Universitário, Quinta da Granja, 2829-511 Caparica, Portugal

**Keywords:** polyhydroxyalkanoate, P(4HB), tissue-engineered substitutes, biomimetic, biocompatible polymers

## Abstract

Polyhydroxyalkanoates (PHAs) are promising biopolymers as an alternative to traditional synthetic polymers due to their biodegradability and biocompatibility. The PHA market is blooming in response to the growing demand for biodegradable and environmentally friendly plastics. These biopolyesters are produced and degraded by a variety of microorganisms, making them environmentally friendly, while offering benefits such as biocompatibility (when adequately processed) and biodegradability. Their versatility extends to various areas, from biomedicine to agriculture and composite materials, where they pave the way for significative innovations. In the field of regenerative medicine, some PHAs have key applications, namely in vascular grafts, oral tissue regeneration, and development of self-healing polymers. In addition, PHAs have the potential to be used in the creation of dental implant materials and dental medical devices. PHAs can also be used to encapsulate hydrophobic drugs, providing an approach for more targeted and effective treatments. To summarize, PHAs open new perspectives in the field of medicine by improving drug delivery and offering ecologically biocompatible solutions for medical devices. The aim of this review is to present the medical and dental applications of PHA, their advantages, disadvantages, and indications.

## 1. Introduction

Polyhydroxyalkanoates (PHAs) represent a class of biodegradable polymers that have gained significant attention in the medical field due to their versatile properties and potential applications. The PHAs are naturally occurring aliphatic polyesters synthesized by various microorganisms as intracellular carbon and energy storage compounds [1].

The green perspective of PHAs stems from their bio-based origin and biodegradable properties, positioning them as eco-friendly alternatives to petroleum-derived plastics. Studies have emphasized the compostability and minimal ecological footprint of PHAs as compared to conventional plastics, aligning with sustainability and environmental responsibility principles [2]. Furthermore, their ability to interact well with biological systems, to break down naturally, and the possibility of adjustable physical properties make them highly favourable options for a wide range of medical uses, such as creating new tissues, delivering medications, and wound healing [3].

**Figure 1:**
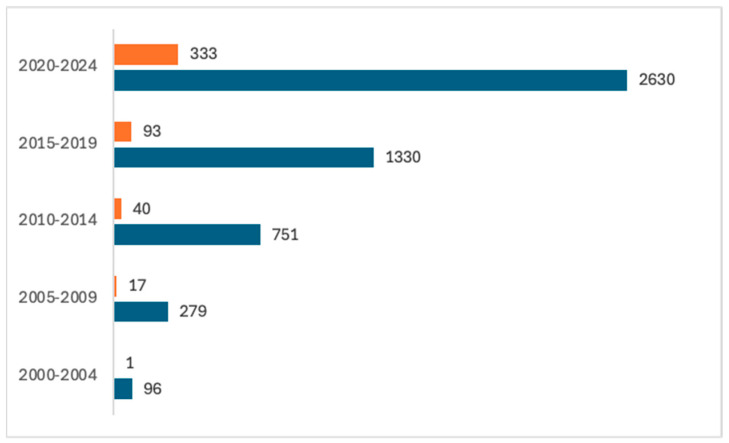
Number of documents found in Scholar Google (dark blue) and Scopus (orange) databases using “dentistry” and “polyhydroxyalkanoate” for the search in “all fields” [4,5].

Recently, research on exploring the potential of PHAs in dentistry is increasing. Figure 1 shows the evolution, in the last 25 years, of the number of documents in Scholar Google and Scopus databases retrieved using “dentistry” and “polyhydroxyalkanoate” for the search (http://www.scopus.com, https://scholar.google.com, accessed 10 October 2024).

The unique characteristics of PHAs, such as their ability to replicate the physical characteristics of organic tissues and their breakdown into harmless substances, make them particularly attractive for dental applications. From sutures to scaffolds for tissue regeneration in oral surgery, PHAs offer a sustainable and biocompatible alternative to traditional materials used in dentistry [6]. This review presents the state of the art on applications of polyhydroxyalkanoates in medicine, gathers current research, and highlights their potential and current limitations for further use in dentistry.

## 2. Overview on Polyhydroxyalkanoates

The increasing buildup of petrochemical plastic waste in the environment is becoming a significant worry. This issue is further compounded by the fact that a large percentage of petrochemical plastics end up in landfills or pollute the environment, contributing to global plastic pollution [7,8]. Some biodegradable PHAs have properties similar to conventional plastics, standing out as a possible alternative. Unlike traditional plastics, polyhydroxyalkanoates are biopolymers developed and broken down by a diverse range of microorganisms, which accumulate within microbial cells, mostly under conditions of nutrient imbalance [9].

Microorganisms produce PHAs from sugars or fatty acids, generating cytoplasmic inclusions that are used by cellular structures to store energy in granular forms. The investigation of poly(3-hydroxybutyrate) (PHB) can be traced back to 1925, when Lemoigne, a French scientist, discovered PHB in *Bacillus megaterium* [10]. Subsequently, over 300 different microorganisms, encompassing both Gram-negative and Gram-positive bacteria, have been acknowledged as PHA producers, growing in anaerobic as well as aerobic environments (namely, *Alcaligenes latus*, *Azotobacter vinelandii*, *Bacillus subtilis*, *Burkholderia sacchari*, *Cupriavidus necator*, *Haloferrax mediterranei*, *Pseudomonas aeruginosa*, *P. mendocina*, *P. putida*, *P. oleovorans*, as well as several methylotrophs, and recombinant *Escherichia coli* strains) [11]. Since 1925, multiple forms of PHAs were discovered and are available in sufficient quantity for different applications. Some of the commonly known ones include PHB, poly(4-hydroxybutyrate) (P(4HB)), copolymers of 3-hydroxybutyrate and 4-hydroxybutyrate (P(3HBco4HB)), polyhydroxybutyrate-co-valerate (P(3HBco3HV)), and poly(3-hydroxybutyrate-co-3-hydroxyhexanoate) (P(3HBcoHHx) [12].

PHAs have desirable thermal and mechanical characteristics, break down naturally, and, when properly processed, can interact well with living organisms, showing no carcinogenic effects. PHA has a significant level of structural variety since many hydroxyalkanoate monomers are available. Given these characteristics, this particular family of polymeric substances has significant practical worth in the field of healing and regenerating tissue [13].

### 2.1. Types of PHAs

**Figure 2:**
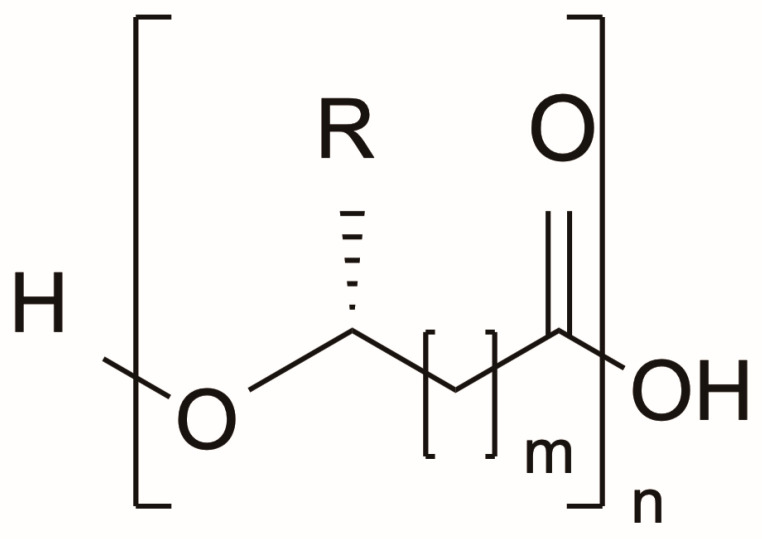
General chemical structure of polyhydroxyalkanoates. The “n” represents the number of monomers in the polymeric chain, and “m” depends on the number of carbons in each monomer.

The basic structure of a PHA polymer molecule, an aliphatic polyester, consists of n (between 100 and 30,000) monomeric units with an R side chain, linked by ester bonds (Figure 2). The side chain (R) can be a saturated, unsaturated, modified, or branched alkyl group, depending on the molecule [14].

The PHAs may be classified into three categories according to the amount of carbon atoms present in the monomers [1]:

- Short chain length PHA (scl-PHAs) consist of monomers containing less than five carbon atoms;
- Medium chain length PHA (mcl-PHAs), ranging from five to fourteen carbon atoms; and
- Long chain length PHA (lcl-PHAs) with over fourteen carbon atoms; lcl-PHAs are rare and have been investigated to a lesser extent.

The type and microstructure of the produced PHA is partly related to the C-sources used for biosynthesis. Homopolymers, block or random copolymers, and even terpolymers can be synthesized [15].

Currently, more than 150 distinct monomers have been identified for the biosynthesis of PHAs, which makes them the most extensive category of natural polyesters, and the most frequently synthesized bacterial biopolymers. These polyesters include short-chain monomeric components, such as 3-hydroxybutyrate (3HB), as well as medium-chain length monomeric components like 3-hydroxyoctanoate (3HO) and 3-hydroxydecanoate (3HD) [16].

### 2.2. PHAs’ Properties

Depending on polyhydroxyalkanoates’ composition, they can present different properties that influence their suitability for a wide range of applications.

#### 2.2.1. Mechanical Properties

Carbon-chain length is one among the parameters that affects a PHA’s mechanical strength, structure, and composition. Several types of PHAs exhibit varying mechanical properties, and adjustments can be made through compounding or synthetic copolymerization to suit specific applications [17].

PHB, an scl-PHA, is known for its rigidity and high strength, with a tensile strength exceeding 40 MPa and a Young’s modulus of 3.5 GPa [17]. However, PHB has limitations such as insufficient processing speed, accelerated deterioration, and limited stretching ability. To address these drawbacks, it is possible to produce copolymers, by incorporating monomers like 4HB, 3HP (3-hydroxypropionate), and 3HV, to decrease the level of crystallinity, enhance flexibility, and slow down the ageing process [18].

P4HB is a thermoplastic polymer and exhibits high ductility. It has a maximum elongation at the point of rupture of 1000%, which makes it one of the most elastic PHA homopolymers synthesized so far [19]. It received FDA approval in 2007 for manufacturing surgical sutures [20].

The addition of different hydroxy acids to the copolymerization of 4HB may expand the variety of characteristics that can be achieved, allowing for the creation of resorbable elastomer compounds with a broad spectrum of durometer hardnesses. The improvement of these new elastomers can lead to applications in absorbable medical devices [20].

In general, mcl-PHAs have lower tensile strength, greater elongation at break, and more elasticity than scl-PHAs. The mcl-PHAs, as explained earlier, are copolymers consisting of monomers such as 3-hydroxyhexanoate (HHx) or 3-hydroxyhexanoate (3HO). The tensile strength and the elongation at break of the most common mcl-PHAs has been reported to range from 5 MPa to 16.3 MPa, and from around 300% to 850%, respectively [21].

Recognising that PHAs’ mechanical properties are still scarcely studied, Bejagam and co-workers (2022) used a molecular dynamics simulation to predict Young’s modulus and yield stress from the number of carbon atoms in the side chain and in the polymer backbone, as well as different side chain functional groups [22].

Due to their versatility, PHAs have a broader range of applications compared to other polymers such as poly(ε-caprolactone) (PCL), polylactic acid (PLA), and polylactic-co-glycolic acid (PLGA). Moreover, the mechanical robustness of PHA may be modified by copolymerization or compounding following the demands of certain targeted settings, making it a flexible substance for many applications [23].

#### 2.2.2. Thermal Properties

The melting temperature (Tm) and glass transition temperature (Tg) of the crystalline phase are often used to represent the thermodynamic characteristics of PHA. The side chain length in PHA molecules has been shown to influence these thermodynamic properties [24]. For instance, P3HB (a polyester molecule formed by 3HB monomers, which has a methyl side chain) has a rather high degree of crystallinity, ranging from 60% to 80%. It has a Tm of around 170 °C and a decomposition temperature (Td) of roughly 200 °C. The narrow difference in temperature between Tm and Td leads to inadequate thermal stability throughout the processing phase. To address this, modifications, such as incorporating other monomers, are often made to enhance thermal stability. Chuah et al. (2013) showed that poly(3-hydroxybutyrate-co-3-hydroxypropionate-co-5-hydroxyvalerate) (P(3HBco3HPco5HV)) with 5HV content ranging from 1 to 32 mol% exhibited low Tm, reduced crystallinity, and elastomeric behaviour [25].

On the other hand, mcl-PHAs usually have a lower level of crystallinity, often about 40%. The Tg of mcl-PHA may exhibit a variety of values, spanning from −52 °C to −25 °C at ambient temperature. Additionally, Tm can vary from 38 °C to 80 °C. Because of the low Tg of mcl-PHA, it exhibits elastic characteristics akin to rubber when exposed to suitable temperatures. When the temperature exceeds Tm, mcl-PHAs undergo a transformation into an amorphous state characterized by its viscous characteristics [26].

In summary, the side chain length in PHA molecules significantly influences their thermodynamic properties, and modifications, particularly in mcl-PHA, can result in materials with unique elastic and viscous properties at different temperature ranges. These characteristics have implications for the processing, applications, and performance of PHA-based materials in various conditions [27].

Research has shown that the value of Tm generally decreases as the length of the side chain carbon increases, whereas Tg grows correspondingly. This leads to differences in thermodynamic properties between scl-PHA and mcl-PHA [28].

Table 1 presents information on scl- and mcl-PHAs’ thermal and mechanical properties. It is, however, important to highlight that the reported values can slightly change according to the molecular weight and polydispersity index for each production batch.

#### 2.2.3. Biodegradability

PHAs exhibit unique biological properties, and one of their distinctive features is their degradability in various environments. PHAs can undergo degradation through the action of mixed microbial populations present in different settings such as rivers, seawater, soil, and other environments. This degradation process can occur under both aerobic (oxygen-rich) and anoxic (oxygen-depleted) conditions [29]. The microbial communities in these environments play a crucial role in breaking down PHA. Their secreted enzymes, called PHA depolymerases or hydrolases, act on PHA polymers. As a result of this enzymatic activity, PHA is broken down into low molecular weight monomers or oligomers that can then be assimilated by microbial cells as nutrients [30]. In biological systems, PHAs can be degraded by depolymerases, and by enzymatic and non-enzymatic hydrolysis [31]. The PHA degradation is usually slow in human and mammal cells when compared to other biodegradable materials, and studies in vivo have shown that it is immunologically inert [32,33]. In the human body, P(4HB) degrades by hydrolysis into 4HB monomers, which are natural human metabolites, and less acidic (pKa of 4.72) than glycolic acid (pKa = 3.83) and lactic acid (pKa = 3.86) [34].

The degradation of PHA is subject to several factors, and the biodegradation rate is influenced by various parameters. The main factor is the PHA depolymerase enzymes’ affinity for the ester linkages found in PHA polymers. Several factors affect this affinity, and they contribute to the overall degradation process. Some of these factors include the following [35]:

- Monomer type and composition;
- Surface area;
- Molecular weight;
- Polymer conformation;
- Tm;
- Crystallinity; and
- Environmental factors.

As an example, scl-PHAs with a higher melting temperature and lower crystallinity degrade more rapidly than mcl-PHAs with the same characteristics [27].

#### 2.2.4. Biocompatibility

PHAs have been shown to promote the proliferation of many types of tissue cells in vitro, such as chondrocytes, epithelial cells, and fibroblasts. When inserted into live organisms, such as rats, human beings, and rabbits, PHA does not elicit a strong immunological reaction. Multiple studies have shown that PHA has exceptional biocompatibility and may even enhance cellular tissue attachment and proliferation [36]. Moreover, PHA’s biocompatibility includes its physiological and immunological reaction during breakdown. The 3-hydroxybutyric acid (3HB), one of the monomers in the PHA polymeric chain, is a natural metabolite that is formed by the oxidation of fatty acids in the liver of humans. It is worth mentioning that 3HB is inherently found in human blood in quantities that vary between 30 and 100 mg/L. The presence of this natural phenomenon in the body indicates that PHA is easily tolerated and embraced by the human system [37].

The 3HB has additional beneficial effects. It has been found to diminish inflammation, facilitate tissue regeneration, and function as a source of energy for the body during times of necessity, such as during starvation. Ultimately, 3HB is excreted as carbon dioxide [38].

Unlike other degradable polymers such as PLA and PLGA, which may cause the buildup of lactic acid products after being implanted, PHA keeps a stable pH level throughout the breakdown process. The PHA’s property of closely aligning with the normal metabolic processes in the human body makes it very favourable in terms of biocompatibility [36,39].

#### 2.2.5. Non-Carcinogenicity

Non-carcinogenicity is a crucial property for biopolymers intended for use in humans, and understanding the potential effects on cancer cell development is an important aspect of evaluating their safety. In the case of PHA, studies have looked at whether PHA promotes the growth of cancer cells while inhibiting their division [13,40].

Research by Peng and coworkers (2011) provided insights into this aspect by investigating the impact of various PHA types, including PHB, P(3HBcoHHx), and P(3HBco3HV), on rat fibroblasts. The findings indicated that these PHA types did not induce carcinogenesis in the cultured rat fibroblasts. The observed accelerated cell growth on PHA was governed by the regular cell cycle. Furthermore, primary rat osteosarcoma cells cultivated in PHA did not change into tumour morphology; instead, they retained their normal shape [13]. Potential pathways via which PHA enhances tissue regeneration are believed to be associated with the response of tissue cells to 3HB and the interactions between materials and cells mediated by microRNAs. While these findings are promising, it is important to note that there is insufficient information to definitively establish that PHA does not have a cancer-causing impact on all types of cells. As a result, further research to investigate the non-carcinogenicity of PHA across various cell lines will be crucial for the continued development and application of PHA in biomedical contexts [13].

A summary of PHAs’ main properties is presented in Table 2.

### 2.3. PHA Bioproduction

Lemoigne revealed in 1923, at the Institut Pasteur, that aerobic spore-forming bacillus produced 3-hydroxybutyric acid in anaerobic suspensions. In 1927, he extracted a compound from *Bacillus* using chloroform and confirmed it was a polymer of 3HB. However, commercial manufacture of PHB did not begin until the 1960s. The pioneering work of Baptist and Werber at W.R. Grace & Co. (Columbia, MD, USA) resulted in many patents for producing and isolating PHB. Baptist and Werber started to test the use of this polymer to make sutures and prosthetics. Their attempts were halted owing to poor fermentation yields and bacterial residues in the polymer. Additionally, the solvent extraction technique was expensive [45].

The PHAs are produced and stored inside the cells of some bacterial strains, mostly under unbalanced growth conditions, such as a lack of phosphorus, nitrogen, oxygen, or magnesium, with an excess of carbon source [46]. When selecting a microorganism for industrial production of PHA, several factors must be taken into account. These include the microorganism’s ability to use a low-cost carbon source, its rate of growth, polymer production rate, and the maximum amount of polymer it can accumulate based on the substrate. To save costs, it is crucial to generate PHA with high productivity. Various techniques, such as continuous and fed-batch reactor cultivations, have been used to enhance productivity [12,47,48]. The PHA production process optimization may be affected by inhibitory chemicals and variations in waste by-products composition, leading to fluctuations in final PHAs concentration, recovery yields, and content. To prevent these variations, careful studies are performed at bench-scale, before moving on to pilot size testing [49].

Different monomers are biosynthesized from different C-sources. Namely, γ-butyrolactone or 1,4-butanediol can be added to cell cultivations as precursors to obtain 4HB monomers [50]. However, strains such as *B. sacchari*, *C. necator* or *Comamonas acidovorans* will produce the P(3HBco4HB) copolymer instead of the P4HB homopolymer [50,51,52]. To obtain the homopolymer free from other PHAs, recombinant strains are used. Wild-type *E. coli* strains are unable to synthesize PHAs, and, as such, they are a good choice to serve as the host for manipulation of the cellular processes towards recombinant P4HB synthesis [53]. Volumetric productivity is a key parameter for industrial PHA production, and factors influencing this parameter can impact the economics and cost competitiveness of PHAs synthesis via microbial fermentation [54]. Moreover, the application of immersed membrane bioreactors could enable semi-continuous PHA production from organic waste-based volatile fatty acids, enhancing the feasibility of upscaling production [55].

Other alternatives to lower total costs include using by-products as a carbon source, high cell density cultivation strategies, and resort to mixed microbial cultures or osmotic strains under non-sterile conditions [56,57,58,59].

### 2.4. PHA Extraction

The PHA extraction from microbial cells is a crucial step in the production of PHA products. The main options for extracting intracellular biopolymers like PHAs from bacterial cells are based in organic solvent extraction, aqueous extraction, and extraction using osmotic shock [60,61,62]. Other methods include enzymatic cell lysis and mechanical cell disruption, which are being explored to improve the efficiency and sustainability of PHA extraction [63]. Alternatives to chlorinated solvents have been successfully tested [64].

Bioproduced PHAs are extracted from the cell biomass recovered at the end of a bioreactor cycle (in the case of batch and fed-batch processes) and the resulting purified product is a mixture of polymer chains with different molecular weights and polydispersity indices. Moreover, when different C-sources are used, different types of molecules are produced, including co- and terpolymers with different molar percentages of monomeric incorporation. This is particularly important, since PHAs with heterogeneous monomer distribution will result in end products with different mechanical and thermal properties, even for the same average monomer distribution. In the case of co- and terpolymers, one way of obtaining purified fractions with a similar monomeric composition and similar properties is to use solvent fractionation [15,65].

The choice of the extraction methods depends on factors like the bacterial strain, PHA content, desired purity, and cost considerations.

## 3. Medical Applications of PHA

In the next sections, an overview on PHA in medical implants (Section 3.1) and in medical devices (Section 3.2) is presented.

### 3.1. PHA as Material for Medical Implants

In the context of medical devices intended for prolonged interaction with human tissues, the primary requirement for a material to be deemed biocompatible is its ability not to harm those tissues. This is typically achieved through chemical and biological inertness [66,67].

In recent years, in vivo degradable implant materials, including PHAs, became increasingly attractive. Using degradable implant materials like PHAs can avoid a second surgery to remove the implant and the potential side effects of permanent implantations [31].

The PHA has attracted considerable interest in biological tissue healing because of its varied structure and excellent physicochemical qualities. Its applications span across soft and hard tissue regeneration, including heart valve, nerve repair, and organ regeneration, as well as bone, vascular, and cartilage tissue engineering [68].

Due to their elastic properties and flexibility, mcl-PHAs are predominantly utilized in soft tissue sectors. These materials are often used for wound dressings, sutures, cardiac patches, cartilage tissue, heart valves, and nerve conduits, applications where flexibility and elasticity are crucial [69,70]. The capacity to adjust the mechanical characteristics of PHAs by creating blends or copolymers has led to the development of composites with enhanced rigidity, making them appropriate for applications such as cartilage tissue [71].

On the other hand, PHB, an scl-PHA, is used in applications involving hard tissues, like bone implants, since it has the required mechanical stiffness to create effective degradable scaffolds with suitable mechanical and physical qualities. Several composites and blends of PHA have been created in this sector [72].

Extremely vascularized bone tissue with remarkable regenerative capabilities can naturally heal minor fractures without surgical intervention [17]. However, significant bone abnormalities, especially those caused by the removal of bone tumours and severe fractures, need surgical treatment. When faced with these situations, many methods are used, such as allografts, xenografts, autografts, or bone implants made from biomaterials [71]. The PHAs have been extensively studied for their applicability in engineering bone tissue owing to their remarkable rates of biodegradation and improved mechanical characteristics.

Recently, researchers have created PHA films with antimicrobial properties specifically for use in bone regeneration. The combination of poly(3-hydroxyoctanoate-co-3hydroxydecanoate-co-3-hydroxydodecanoate) (P(3HOco3HDco3HDD)) and PHB-based blends with selenium–strontium–hydroxyapatite was used to provide antibacterial characteristics without the use of antibiotics. Hydroxyapatite was added to promote the incorporation of tissue into bone and enhance the attachment and growth of osteoblasts. These films demonstrated significant antibacterial efficacy against *Escherichia coli* 8739 and *Staphylococcus aureus* 6538P resulting in the production of diverse films with distinct mechanical characteristics [73].

Basnet and coworkers conducted research in 2021 where they created fibrous scaffolds made of poly(3-hydroxyoctanoate-co-3-hydroxydecanoate) (P(3HOco3HD)) and poly(3HB) utilizing pressurized gyration. These scaffolds were intended for use in bone, nerve, and cardiovascular tissues. The osteoinductive capabilities of composite PHB/fibers of HA were assessed utilizing Chorioallantoic Membrane Studies (CAMs). In addition, the composites were inserted under the skin of mice with weakened immune systems to evaluate the development of bone tissue, the growth of blood vessels, and the invasion of host tissue. The PHB fibers incorporated with HA and implanted with Stro-1+ human bone marrow stromal cells (HBMSCs) exhibited the greatest degree of vascularization, a considerably higher quantity of vessels as compared to PHB fibres seeded with HBMSCs alone, and increased collagen deposition [74]. These positive outcomes highlight the potential of PHA composites for hard tissue engineering applications, given their osteoinductive properties and ability to promote angiogenesis.

The composite scaffolds made of HA and PHB exhibited the capacity to stimulate a sustained bone tissue adaptation response without causing any foreign body reaction. Studies in vitro have shown that the HA/PHB scaffold facilitated the attachment, differentiation, and growth of HBMSCs and human maxillary osteoblasts (HOBs). The PHB/HA scaffolds, which were covered with type I collagen, stimulated the development of new bone tissue in nude mice models. The high levels of osteocalcin expression throughout the implantation period validated the osteogenic properties of the cell-seeded PHB/HA scaffolds, while the control group did not exhibit any discernible osteocalcin expression or osteoinduction [75].

Incorporating a precise quantity of nano-sized Bioactive Glass (nBG) into the PHB scaffold resulted in a notable increase in the growth of human osteosarcoma cells (MG63) [76]. This also led to better ability to support bone formation and increased alkaline phosphatase (ALP) function [77]. Hajiali conducted studies to assess the viability of utilizing PHB/nBG nanocomposites as scaffolds for bone tissue engineering. After submerging the PHB/nBG scaffolds in simulated bodily fluid (SBF) for a period of one month, a thin layer of HA mineralization developed on the surface. This process improved the potential of the scaffolds to facilitate osseointegration and bone restoration [78].

Hydrogels were combined with PHB to create bioactive osteogenic scaffolds. Volkov et al. created Alginate (ALG) hydrogel-based PHB/HA scaffolds by a salt-leaching process and using 3-D printing. The scaffolds provided strong support for the hydrogels, and their interconnected pore architectures promoted the proliferation of Mesenchymal Stem Cells (MSCs) and stimulated the development of bone tissue. The use of hydrogel-based PHB/HA composite scaffolds laden with MSCs successfully repaired radial bone defects in rat parietal bone at a high rate (94%) on day 28. This finding underscores the promising capabilities of bioactive polymer scaffolds in bone tissue engineering [79].

Another promising material for bone tissue engineering, P(3HBco3HV), was widely mixed with bioactive substances. The P(3HBco3HV) demonstrated mechanical compressive strength that is similar to that of human bone, and it enhanced the experimental bioactivity of the PHA scaffold. Animal tests have shown that the P(3HBco3HV)/HA composite significantly increased the thickness of newly produced bone from 130 μm to 770 μm over six months. This suggests that the composite has excellent properties for enhancing bone structural repair [80].

The MSCs and osteoprogenitor cells are stimulated to differentiate into osteogenic tissues by adenosine, another bioactive substance combined with P(3HBco3HV). Zhong and collaborators (2019) conducted a thorough investigation of the osteogenic potential of composite nanofibers by integrating adenosine molecules in P(3HBco3HV) electrospun nanofibers, both in laboratory conditions and in living organisms. The electrospun fibers made from P(3HBco3HV) and loaded with adenosine showed significant efficacy in promoting bone regeneration when used to treat skull deformities in rabbits [81].

In addition, Cool et al. (2007) conducted research to examine the osteogenic characteristics of P(3HBco3HV) combined with β-tricalcium phosphate (β-TCP) and calcined hydroxyapatite (CHA), respectively. The addition of nanoscale-reinforced phases to P(3HBco3HV) resulted in enhanced osteogenic characteristics and reduced inflammatory response [82]. To considerably promote the creation of apatite, Chernozem et al. (2019) presented a unique scaffold that combines PHB and P(3HBco3HV) with mineralized piezoelectric bodies made of calcium carbonate [83].

Minerals have the potential to greatly enhance the development of apatite surrounding PHB/P(3HBco3HV) scaffolds, hence promoting the creation of bone tissue. These investigations have introduced a novel therapeutic approach for using P(3HBco3HV) polymers in the regeneration of bone tissue [84].

### 3.2. PHA in Medical Device Manufacturing

The PHA homopolymers like PHB, P(4HB) and P(3HO); copolymers such as P(3HBco3HV), P(3HBco4HB), and P(3HBcoHHx); and composites including these polymers were used in the development of devices including repair devices, sutures, repair patches, slings, orthopedic pins, adhesion barriers, cardiovascular patches, and stents [85].

#### 3.2.1. Heart Valves

Surgical replacement of heart valves, whether biological, artificial, or mechanical, is a highly efficient treatment approach for treating heart valve diseases. However, these valves often do not expand and have limited durability, which may result in thrombotic issues caused by rough surfaces that encounter blood. Consequently, Heart Valve Tissue Engineering (HVTE) has emerged as a crucial strategy for addressing these challenges under dynamic conditions. Recent studies highlight PHA as a very suitable substrate for HVTE, showing the use of a P4HB scaffold to generate a HVTE [86].

A tri-leaflet heart valve scaffold was created for the first time by Sodian et al. (2000) with the thermoplastic PHA polymer P(3HBco4HB). This scaffold, produced using stereolithography, facilitated the introduction of ovarian artery vascular cells, enabling the coordinated pulsation of the three-leaflet valves. The cells thrived and effectively generated an extracellular matrix (ECM) when exposed to pulsatile flow [87]. Later studies included replacing the original pulmonary valve in lamb animals with sheep carotid artery vascular cells injected onto the P4HB heart valve scaffold. The findings indicated that the P4HB heart valve scaffold was enveloped by healthy tissue without any blood clot formation, and the valve exhibited no notable backflow within 120 days after the surgery. The pathological results revealed the existence of fibrous tissue with glycosaminoglycans as the ECM in the vicinity of the scaffold. Furthermore, PHA polymers have been used for the purpose of covering or patching heart valves. Decellularized porcine aortic valves that were coated with P(3HBcoHHx) show enhanced resistivity, high tensile strength, and decreased calcification when tested in a living organism [88].

Motta and coworkers (2019) illustrated the potential to create a tissue-engineered heart valve using human cells. Furthermore, they introduced a unique valve design incorporating the Valsalva sinuses’ anatomical characteristics and confirmed the practicality and safety of Transcatheter Pulmonary Valve Replacement using readily accessible tissue-engineered human cell-derived valves in experiments conducted on sheep [89].

A different example pertains to a heart valve with three leaflets that has an elevated level of porosity. This valve has a P4HB coating and is made of PGA. Perry et al. (2003) isolated and cultured Bone Marrow-Derived Mesenchymal Stem Cells (BMSCs) and then seeded them onto the PGA/P4HB composite scaffold. This process led to the development of a tissue construct that exhibits biomechanical properties comparable to natural heart valves. The diameter of the valve scaffold increased in sheep after implantation for 20 weeks, suggesting the possibility of future expansion [90]. The natural flexibility and malleability of P4HB enabled it to adjust to tissue development, while stem cells multiplied and specialized on the scaffold surface, creating an even coating of fused cells and extracellular matrix. Baghdadi et al. (2018) created a versatile cardiac patch utilizing PHO that has a mechanical strength similar to that of myocardial tissue [91].

The PHB composite scaffolds demonstrated a reduction in the inflammatory response when compared to other materials used for cardiac repair in infarcted rat hearts. Their induction of considerable angiogenesis renders them more advantageous for the healing and remodeling of heart tissue [92].

#### 3.2.2. Nerve Engineering

The manufacturing technique for nerve tissue aims to repair damage in the peripheral nervous system. It is recognized that natural polyesters are useful biomaterials for treating diseases of the peripheral nervous system. Biocompatible materials or biodegradables of biological origin are suitable for nerve path applications. The mcl- and scl-PHAs have been utilized to treat nerve tissue and regenerate nervous cells [93]. Contemporary methods for repairing peripheral nerves concentrate on creating substitutes for nerve autografts, such as nerve tubes, guides, or conduits. These substitutes aid in the growth of axons and may span wider gaps in nerves [94].

Studies have indicated that P(3HBcoHHx) is effective in Schwann cell regeneration, particularly when combined with PHB [95]. Additionally, blending P(3HBcoHHx) and poly (DL-lactide) has shown promise for creating an effective nerve path [18]. Blended P(3HBcoHHx) has demonstrated favourable outcomes, which can be attributed to the reduced crystallinity compared to pure P(3HBcoHHx). Another noteworthy approach involves blending PHBV microtubules with PLGA to enhance the flexibility of films used in nerve tissue engineering [96].

Researchers have explored blended composites of P(3HBco3HV) with collagen for nerve tissue regeneration. Mixed films or microspheres of P(3HBco3HV)/collagen are preferred over pure polymers due to their higher proliferation ability for nerve cells [97]. Masaeli et al. produced electrospun scaffolds by combining PHB and PHBV nanofibers for the redevelopment of myelinic cells [98].

#### 3.2.3. Sutures

The skin can naturally regenerate, and minor injuries heal independently [72]. However, in some instances, such as wounds with varying depths, severity, microbial invasion, and depending on the patient’s health, external interventions like sutures, wound dressings, and, for extensive defects, skin grafts or tissue-engineered skin may be necessary [99].

Sutures can be absorbable or non-absorbable, require materials that are antibacterial, biocompatible, possess high tensile strength, easily sterilizable, and can be securely fastened [100].

The use of PHAs in medical applications, particularly in sutures, has been well-researched and validated. The PHAs have proven to be advantageous for sutures, with P(4HB), PHB, P(3HBco3HHX), and P(3HBco3HV) [3]. The FDA granted approval, in 2007, to P4HB, commercially known as TephaFLEX^®^ (Tepha, Inc. Lexington, MA, USA), as a suture material. The P(4HB) stands out for absorbable sutures due to its less acidic degradation product compared to PGA and PLLA, as well as its faster degradation compared to PLLA, PCL, and other PHAs like PHB [101]. Subsequently, two more PHA-based suture materials MonoMax^®^ (B. Braun, Tuttlingen, Germany)) and Phantom Fiber™ (Tornier Inc, Bloomington, MN, USA), both using P(4HB), received FDA approval [100]. Monomax^®^ is an ideal thread for closing the abdominal wall due to its exceptional characteristics. Furthermore, Monomax^®^ exhibits a much greater tear resistance in comparison to standard long-lasting tear-resistant sutures. It also provides physical support to the abdominal wall for six months, hence ensuring optimal performance [102].

Suture research employing PHAs was focused on altering P(3HBco3HV) and P(3HBco3HHx) for suture applications [3]. An experiment combining the low molecular weight polymers P(3HBco3HHx) and PLLA at a ratio of 20:80 was conducted to produce films. The results showed enhanced mechanical capabilities, greater toughness, and faster degradation. This combination has been recognized as an exceptional option for producing “resorbable medical sutures” [103].

#### 3.2.4. Stents and Vessels

A major issue linked with traditional metallic stents used in medical applications is the potential for restenosis, which involves the re-narrowing of the treated artery. To tackle this challenge, there is a need for the advancement of biodegradable stent technology. These stents are designed to perform their function of widening blocked arteries and subsequently degrade naturally, reducing the risk of restenosis. Recent advancements in this field include using biodegradable stents made from PLLA/P(4HB), which demonstrated encouraging outcomes when inserted into a porcine model. More precisely, when an oral atorvastatin medication was used in combination with these stents, there was a reduced level of narrowing compared to situations when permanent 316L (stainless steel) stents were used [104].

This progress indicates the potential for a better stent design, with PHAs emerging as an auspicious material for such applications. Due to their flexibility, which allows them to expand in response to blood flow pressures, elastomeric mcl-PHAs are especially attractive for blood arteries. Recently published research has shown that P(3HO) may be effectively melt-processed for tube extrusion by using bacterial cellulose nanofibers. The enhanced thermal and mechanical qualities obtained indicate a strong potential for using tissue-engineered blood arteries in live organisms [105].

#### 3.2.5. Wound Dressing

Wound dressings are crucial in promoting the process of healing for both chronic and acute wounds, providing coverage and protection to the injured area [106]. The selection of materials for wound dressings is essential, and they should be easy to use, non-adherent, sterile, and capable of preventing bacterial infections. The selected material must also support angiogenesis, facilitate gas exchange, maintain a moist environment, and promote the proliferation and migration of keratinocytes, fibroblasts, and epidermal cells. PHAs exhibit considerable potential for use in skin tissue engineering and wound dressings. Previous studies have demonstrated that fibroblasts and keratinocytes stick and multiply more effectively on PHA-based materials than other synthetic polymers [107].

Laboratory experiments conducted with murine fibroblasts have demonstrated that nanofibers made of P(3HBco4HB) incorporating collagen peptides promote the adhesion and growth of cells [108]. Animal studies conducted on rats with full-thickness open excision-type skin wounds have shown that the utilization of P(3HBco4HB)/collagen nanofibers substantially accelerates wound healing, achieving a closure rate of 98% compared to the control treatment with gauze, which only achieved 63% healing [109].

GalaFlex™ Scaffold Collection, a range of 100% P(4HB) products by Tepha (Inc. Lexington, MA, USA) is already being commercialized, and is designed for soft tissue repair support in plastic and reconstructive surgery [20,110].

### 3.3. PHA as Drug Delivery Systems

PHAs find applications in drug delivery due to several advantageous properties [111,112]. One key advantage is their tunability, which is achieved through various production methods and allows for the controlled release of therapeutics over specific periods. Moreover, PHAs can be adjusted to focus on certain regions inside the body. Drug-delivery systems include several methods such as injectable nanoparticles for oral and pulmonary administration, transdermal materials and devices, and drug-delivery implants [113]. PHAs have been employed in producing drug-delivery devices, incorporating diverse methods such as films, patches, and even prototypes [114]. However, the FDA has not approved any PHA-based nanomedicines for clinical applications, and their use is still limited to the experimental stage [115].

The PHAs’ tunable biodegradability is particularly valuable for these applications. This property allows PHA-based drug-delivery systems to degrade at a controlled rate, aligning with the desired release profile of the therapeutic agent. With their versatility, PHAs can be used in various drug-delivery systems such as injectable nanoparticles, transdermal patches, and other prototypes, allowing for tailored release kinetics and targeted delivery within the body. Biodegradability is particularly valuable for these applications [116].

One major challenge in the treatment of cancer is balancing the anti-cancer effects while minimizing damage to healthy cells. A solution to this challenge involves developing non-toxic treatments targeting cancerous cells. Surface erosion observed in scl-PHAs makes them an excellent candidate for drug delivery. However, issues such as crystallinity and high hydrophobicity can result in the rapid release of medications without polymer degradation. In contrast, mcl-PHAs, with their low crystallinity and low temperature, have been reported to provide better drug-delivery outcomes [117,118].

A recent development in cancer treatment involves combining PHAs as carriers of hydrophobic photosensitizers (such as phthalocyanines, porphyrins, and related substitutions) for photodynamic treatment [119]. These photosensitizers demonstrate a strong propensity for accumulating in the membranes of intracellular organelles like mitochondria [120]. Research has shown that porphyrin sensitizers induce an antineoplastic impact in myelocytic cell lines similar to K562 cells after laboratory photodynamic treatment protocols [121].

Kilicay et al. (2024) conducted a study on the utilization of salicylic acid (SA) placed into PHB nanoparticles (NPs) to enhance the effectiveness of encapsulating caffeic acid and folic acid (FA) for the treatment of breast cancer, with the aim of increasing both the encapsulation efficiency and anti-cancer activity. The nanoparticles (NPs) were generated via the process of solvent evaporation and then analysed using a range of different techniques. The findings indicated that the nanoparticles had excellent stability for a duration of 30 days, while also maintaining a continuous release for a period of 25 days. In summary, the research determined that FA–Caff NPs have great potential as a carrier system for the treatment of breast cancer [122].

According to another study conducted in 2020 by György Babos, dual drug-loaded nanotherapeutics have been found to effectively address medication resistance and mitigate negative effects caused by some drugs. The study utilized a specialized poly([R, S]-3-hydroxybutyrate) to co-encapsulate Doxorubicin and sorafenib through an emulsion-solvent evaporating technique. The nanoparticles were conjugated with poly(ethylene glycol) (PEG) to enhance the properties of medication release and make them stealthier. The generated PHB-sorafenib-doxorubicin nanoparticles were 199.3 nm on average, and following PEGylation, they increased their average diameter to 250.5 nm. While the attachment of PEG resulted in a minor reduction in the production of nanoparticles and the effectiveness of drug encapsulation, it had a positive effect on the release of Doxorubicin. Specifically, the drug was released more rapidly in a tumor-specific acidic environment compared to blood plasma. Although the PEG connection slowed down the release of both medications, sorafenib’s release still occurred at a much higher pace, with an enhanced initial burst, compared to Doxorubicin. In vitro, the PEG–PHB copolymer exhibited more favourable drug release kinetics compared to the newly discovered PEGylated poly(lactic-co-glycolic acid) nanoparticles that contained the same medicines [123].

Films of P(3HBco4HB) with 26% 4HB and P(3HBco3HV) with 18% 3HV molar incorporation have been tested to assess the possibility of obtaining a combined effect of two antibiotics (ceftazidime, doripenem), an antiseptic (chlorhexidine), and a tissue regeneration stimulator (actovegin) on cellular regeneration processes [124]. After successful drug release kinetics assays, antiseptic properties against *E. coli* and *Staphylococcus aureus* experimental studies, and cell regeneration activity assessments, Murueva et al. (2024) concluded that the tested drug depot films are potentially useful for skin regeneration and will use this study as the basis for in vivo testing [124].

Hyaluronic acid-based microneedles were recently tested in mice for the application of Ritlecitinib (a new drug for fighting hair loss) encapsulated in PHAs nanoparticles, together with vascular endothelial growth factor [125]. For this study, the terpolymer P(3HBco3HVco3HHx) containing 90 mol% of 3HB, 3 mol% of 3HV and 7 mol% 3HHx, produced by Bluepha Co., Ltd. (Beijing, China), which is slowly biodegradable in vivo, was chosen. This minimally invasive transdermal drug-delivery system appears to be an effective therapeutic approach for treating androgenetic alopecia, promoting improved hair regeneration [125].

## 4. Use of PHA in Dentistry

In dentistry, PHA might be used in various ways, such as developing biodegradable materials for dental applications, including temporary dental devices, sutures, or other materials used in oral and maxillofacial surgery. The biodegradable nature of PHA could be advantageous in specific dental applications, especially when temporary support structures are needed and can naturally break down over time [126]. Table 3 provides an overview of the in vivo dental applications of PHA.

### 4.1. PHA as a Healing Scaffold

The PHA has exceptional mechanical characteristics, biocompatibility, and degradation rates, making them very suitable for tissue engineering purposes. The PHB, P(3HBco3HV), P4HB, and their composites, have been used in diverse medical devices and bone tissue engineering. The PHA scaffolds possess a mechanical compressive strength comparable to that of human bones, elicit a delayed inflammatory response, and promote more mineralization. The PHA has been used in several formats, such as micro-grooved membranes, aligned nanofibers, and composite materials filled with carbon nanotubes. The PHB has shown its ability to promote the development of human mesenchymal stem cells into bone tissue. Nevertheless, the high crystallinity, extended degradation period, and hydrophobic properties of the material restrict its potential uses [130]. Various research investigations have made efforts to alter the material characteristics of PHB by combining it with other substances, such as a 50:50 mixture of PHB with P(3HBco3HV), electrospun nanofibrous scaffolds, and electrospun nanofiber scaffolds made from a blend of PHB and cellulose acetate. The PHA exhibits a significant in vitro cell adhesion, sustained proliferation for a duration of 10 days, enhanced differentiation, osteoblast regeneration, and provides cellular protection against stressors encountered during injection. When PHB scaffolds are covered with PHA granule binding protein coupled with RGD peptide, they exhibit enhanced adhesion, proliferation, chondrogenic differentiation, increased synthesis of extracellular matrix, and dramatically improved cartilage-specific characteristics [130].

The PHA have proven effective in various tissue engineering areas, but their potential in regenerating oral tissue is largely unexplored. The oral cavity is home to many bacteria and microbes that can harm systemic health, leading to dental and periodontal conditions. Soft tissue deficiencies or post-implantation scenarios increase the risk of infections; therefore, there is a need for scaffolds with antibacterial properties to decrease the chances of wound infections and ameliorate the outcome rate of gingival surgery [12].

Recent studies predominantly target the incorporation of antibiotics into scaffolds; however, limitations such as poor absorption, adverse reactions, and antibiotic resistance prompt the exploration of novel strategies. In addressing these challenges, the proposed solution involves the development of antibacterial scaffold with porosity based on flexible PHA embedded with 3HB and 4HB (PAP34HB). A porous antibacterial scaffold made of P34HB, called PAP34HB, was developed to address complex oral soft tissue defects in healthy male Wistar rats. The scaffold had a unique asymmetrical interconnected porous structure that provides a barrier, which made it superior to commercial collagen membranes (CMs) in terms of pro-healing, antimicrobial, and biocompatibility qualities. Even in the presence of infection, PAP34HB exhibits a significant healing impact on soft tissue anomalies in the mouth [131].

### 4.2. PHA for Oral Bone Regeneration

The PHAs, when used as nanomaterials, demonstrate excellent potential as biomaterials for the development of bone scaffolds as previously explained, especially in combination with other biomaterials including cellulose acetate (CA), PLA, polyL-lactide-co-caprolactone (PLCL), and hydroxyapatite (HA). The P(3HBcoHHx)/P(3HOco3HHx)) and P(3HBcoHHx) with poly-lactide-co-caprolactone (P(3HBcoHHx/PLCL) are examples of PHA blends. Because of the planned micro- and macro-scaffold porosity, PHAs have proved effective in encouraging osteoblast cell adhesion [132].

Combined with HA, these PHA blends result in nanocomposites exhibiting impressive compressive strength, similar to human bones. The PHAs also demonstrate enhanced osteoblast regenerative properties, protect stem cells against tension, promote cell proliferation in damaged tissue areas, and exhibit improved adhesive properties compared to other biomaterials [133].

In a study conducted by Zinn and coworkers (2001), ten dogs underwent the removal of their third and fourth mandibular premolars on both sides of their jaws. The extracted teeth were then replaced with an occlusive barrier made of a poly(3-hydroxybutyrate-co-3-hydroxyvalerate) (P(3HBco3HV)) membrane reinforced with polygalactin 910 (vicryl) fibers. After 8 and 12 weeks, researchers observed that P(3HBco3HV) films were more mechanically sound and elicited a better tissue response compared to other synthetic polymers like PLA and polycaprolactone (PCL) [134].

Membranes made of P(3HBco3HV) were compared to PTFE membranes to assess their tissue reaction post-implantation and to provide a barrier between bone and soft tissue. The study showed that P(3HBco3HV) membranes were superior to polytetrafluoroethylene membranes in promoting tissue regeneration and tolerance. Nonporous P(3HBco3HV) films effectively separated soft tissues and bone until the completion of the tooth transition, typically occurring around 24 weeks. The results showed that P(3HBco3HV) films exhibited superior mechanical characteristics and tissue responsiveness compared to PLA and PCL [135].

Research on P(3HBco3HV) included its use in advanced regenerative dental treatments, including in the treatment of jawbone anomalies. The membranes serve as an obstacle to inhibit the infiltration of soft tissues, facilitate the full regeneration of newly formed bone in defects, and enhance the dimensions of the alveolar ridge. P(3HBco3HV) membranes were employed to study the therapy of mandibular anomalies and to enhance the thickness of the rat jaw. The findings indicated enhanced bone regeneration from 15 to 180 days, resulting in complete space fill with newly created bone within six months [136].

**Figure 3:**
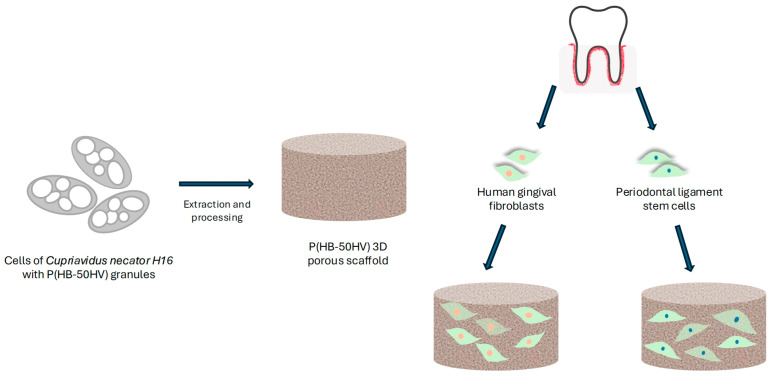
Biomaterial scaffold made from P(HB-50HV) for periodontal tissue engineering adapted from [137].

Three-dimensional porous scaffolds were created utilizing P(3HBco3HV) with a 50% 3HV [137]. The polymeric material, denoted as P(HB-50HV), was bioengineered from the bacterium *Cupriavidus necator* H16, and its potential for in vitro proliferation of dental cells in the context of tissue engineering was assessed (Figure 3).

Compared assessments were performed using scaffolds made from PHB, PCL, and P(3HB-12HV). The polymer films showed a hydrophilic character based on water contact angle studies. All the constructed scaffolds had a significant porosity of 90% and a sponge-like structure [137]. A high porosity structure, with interconnected pore networks, favours the migration, deposition and proliferation of cells, as well as their access to nutrients and oxygen [138,139]. However, mechanical properties must not be compromised due to the excess of void space while the scaffold is still being colonized. In general, the strength resistance should resemble the in vivo natural material mechanical strength [138,139]. Phuegyod’s team discovered unique differences in compressive stiffness in the P(HB-50HV) scaffolds. These scaffolds demonstrated a lower rigidity compared to all other examined scaffolds in both dry and wet environments. Human gingival fibroblasts (HGFs) and periodontal ligament stem cells (PDLSCs) showed strong attachment, increased growth, and maintained a healthy cell shape when grown on the P(HB-50HV) scaffold. The results highlight the outstanding cells compatible with P(HB-50HV) scaffolds, indicating their possible use as biomaterials in stem cell-based treatments and periodontal tissue engineering [137].

Galgut et al. (1991) examined the histological response of P(3HBco3HV) membranes, observing good tolerance and minimal downgrowth of epithelial tissues [140]. Qu and coworkers (2006) compared the degradation behaviour of P(3HBco3HHx), PHB, PLA and blends of P(3HBco3HHx) with PEG implanted subcutaneously in rabbits, while also evaluating the tissue reactions. PHB degraded at a slower pace, followed by P(3HBco3HHx), and then by PLA. For P(3HBco3HHx) blended with PEG, the degradation was faster, however, this blend caused a strong tissue response. The authors also concluded that P(3HBco3HHx) degradation occurred preferably in amorphous zones, creating porous structures for subsequent enzymatic and non-enzymatic hydrolysis [141].

### 4.3. PHA in Controlled Drug Delivery for Oral Disease

Oral mucosa ulceration is a common condition that may develop at any age. Therefore, pain can reduce the quality of life. The current options for treating a medical condition often involve using topical corticosteroids. Unfortunately, these options use poor drug-delivery systems and need to provide adequate contact time for the medication to work effectively [142].

Owji and co-workers (2021) developed a technique inspired by mussels that uses simple polydopamine chemistry to replicate their adhesive functionality. This technique allows for optimal adhesion and locally controlled medication delivery to the affected area. The polydopamine chemistry technique was coupled with PHA as the delivery system to achieve this. The authors focused on producing the PHA via *Pseudomonas mendocina* CH50, which is isolated from the bacterium and transformed into films using solvents. The polydopamine coating was applied by submerging the solvent-cast in a film made from a solution of polymerized dopamine [143].

Periodontitis is defined as a condition affecting the gums and other structures that support teeth. The treatment of periodontal disease focuses on reducing infection by removing germs that cause periodontitis by mechanical cleaning and administering systemic antibiotics. Tetracycline (TC), a versatile antibiotic, is often used in situ with mucoadhesive polymers to treat periodontal conditions because of its ability to combat many types of bacteria responsible for periodontitis [144,145,146,147]. Systemic use of antibiotics at large doses may cause various adverse effects such as gastrointestinal discomfort, hypersensitivity, and an elevated likelihood of bacterial resistance [148]. The concern with systemic antibiotic administration is the potential adverse effects that may impact the patient’s overall health. Hypersensitivity reactions can manifest as allergic responses, while gastrointestinal intolerance may cause discomfort and digestive issues. Additionally, the overuse of antibiotics can contribute to the development of bacterial resistance, reducing the effectiveness of these drugs over time. Researchers and healthcare professionals are exploring alternative approaches to deliver antibiotics directly to the affected site to address these challenges and minimize systemic side effects. Localized delivery methods such as antibiotic-loaded gels, films, or other carriers allow targeted treatment, reducing systemic exposure and associated side effects [149].

In this context, Panith et al. (2016) worked on the synthesis and characterization of drug-delivery vehicles using PHAs for the gradual release of TC in periodontal therapy. Four distinct PHA variations were used to create TC-loaded PHA microspheres. The authors investigated how various features of the microspheres were influenced by using varying molecular weights of polyvinyl alcohol (PVA) as a surface stabilizer and found that the release speed of PHA microspheres was affected by the kind of PHA and the molecular weight of the PVA stabilizer. The research showed that TC-loaded PHB microspheres efficiently killed germs that cause periodontitis, indicating their potential use in curing periodontal disease [150]. The particle size, drug loading, and drug-release profile could be modified depending on the molecular weight of the PVA particle stabiliser, the core PHA polymer type, and the %HV content in PHA copolymers. An increase in the percentage of highly volatile content resulted in a reduction in particle size and lowered encapsulation efficiency. Microspheres with increased crystallinity had a slower drug release velocity, but those with a larger percentage of 3HV content showed a greater initial burst release and quicker drug release. The findings indicated that the TC released from PHA microspheres followed a Fickian diffusion process. The drug loading and release characteristics were affected by the molecular weight of PVA. Low Mw PVA exhibited more adsorption to PHA particle surfaces and increased drug encapsulation efficiency. The research emphasized the potential of PHB as follows: low molecular weight PVA microspheres for extended drug release and strong antibacterial effects against bacteria that cause periodontitis, indicating its suitability for future use in periodontal treatment [150].

### 4.4. PHA in Dental Prosthetics and Devices

One of the most used sutures in dentistry is the synthetic absorbable braided suture Coated Vicryl Rapide^TM^ (polyglactin 910), which consists of a copolymer of 90% glycolide and 10% L-lactide monomers, coated with 90% caprolactone and 10% glycolide and then with a 50/50 mixture of copolymer of glycolide and lactide (polyglactin 370) and calcium stearate [151]. This rapidly degraded (usually it is completely absorbed within 6 weeks), nonantigenic, nonpyrogenic material has similar characteristics to collagen (surgical gut) sutures. However, it can cause mild tissue reaction upon absorption. As described in Section 3.2.3, P(4HB) is currently the only approved PHA for medical use and is commercialized under different trade names. Compared with Vicryl Rapide^TM^, P(4HB) has longer degradation times. Other PHAs are being tested to be used as suture materials. While PHA-based sutures offer a promising alternative to traditional sutures in dentistry due to their biocompatibility, biodegradability, and potential for improved wound healing, there are also some challenges associated with the use of PHA-based sutures in dentistry. One of the main challenges is the high cost of production, which can limit their commercialization and widespread use; additionally, the mechanical properties of PHA-based sutures can vary depending on the composition of the polymer, and they can be affected by the degradation of the polymer and the environment in which they are used [152]. Therefore, further research is needed to optimize the production and properties of PHA-based sutures for use in dentistry [152].

Tooth regeneration remains a major challenge, because, unlike some other parts of the body, teeth cannot regenerate due to age related changes that affect their ability to grow back naturally. To address this gap, dental implants are commonly used in dental practice to replace missing teeth and provide support for fixed or removable partial teeth. The adequacy of the materials used for these dental implants is crucial, in terms of resistance to chemical attack, environmental stress, as well assigning rigidity and thermal stability [153].

At the same time, research into materials that mimic the properties of natural dental enamel represents a promising pathway for the development of more effective dental implants. In this perspective, Gregory et al. (2023), focused on the development of various medical devices, including dental implants, bone materials, and repair patches for osteochondrial tissues. They opted for Fusion Deposit Modeling to design computer-assisted drawings for these devices, before implementing them with a 3D printer to validate their concept. Subsequently, these researchers successfully designed tailor-made dental implants for patients, using precise computed tomography images and applying mirror and mesh fusion techniques to reconstruct the missing parts. The use of materials such as PHAs and PLAs, common bioresorbable medical polymers, has enabled the creation of biocompatible dental implants. These implants are designed to withstand the strain of chewing, preserve the dental structure and harmonize with the aesthetics of other teeth [152].

Another possibility for PHA use in dentistry is the design of surgical guides for dental implants that provides benefits in terms of biocompatibility and precision. Surgical guides printed in 3D, such as those made using Surgical Guide Resin, are sterile and biocompatible, ensuring patient safety throughout dental procedures. The creation of these guides includes numerically calculating patient dental models, precisely planning the placement of implants, and creating a surgical guide using a specific software [154]. Gielisch et al. (2022) conducted a study to investigate the precision of surgical guides made of PLA/PHA and MED610 jet material in the implantation of fully guided dental implants before and after vapor sterilization. The PLA was from a filament by Raise3D Inc (Lake Forest, CA, USA). The MED610 is a solid, transparent PolyJet™ (Stratasys, Eden Praire, MN, USA) material designed for medical applications. The study compares the precision of these surgical guides made by different materials and the difference between the planned and actual implant placements. The goal is to reduce the environmental effect of the medical industry by developing precise and biodegradable materials. After demonstrating levels of precision comparable to those of materials currently used in clinical practice, the research concluded that PLA/PHA surgical guides are a practical and environmentally friendly alternative [155].

To finalize this section, a few examples of patents granted for PHA use in the dentistry field are presented in Table 4.

## 5. Critical Evaluation—Opportunities and Present Limitations

The focus on optimizing bioproduction and isolation techniques is crucial for making PHAs commercially viable alternatives to traditional petrochemical polymers [162]. To achieve PHA suitable concentrations in industrial bioreactors, a wide range of substrates and bacterial strains are used. The cost of producing a PHA is still significantly higher (3–12 times) than traditional petroleum-based plastics [163]. Fifty percent of the total cost of manufacturing is attributed to the price of the carbon source [164]. Several research works focus on selecting industrial and agricultural by-products as a supply of carbon to lower the cost of producing PHA [15,165,166]. When attempting to produce specific co-polymers, a mixture of homo- and copolymers is frequently obtained, hence, cost effectively separating polyesters and copolyesters with different chemical compositions and physical characteristics is another research goal [167].

The majority of PHA materials on the market are not meant to be used in implant procedures. Rather, they want to penetrate the low-end recyclable or biodegradable packaging industry [168]. In fact, the optimization of PHA bioproduction for each targeted application requires a multidisciplinary approach. Microbiology, biochemistry, biomaterials, and bioengineering researchers need to closely collaborate with health professionals (surgeons, physicians, dentists, among others) to focus their research on PHA’s specific applications regarding human health. The PHA’s tuneable properties, which depend on microbial strains, carbon sources, and specific enzymes’ activity, open possibilities for a broad variety of purposes.

As previously described, the PHA is a versatile biodegradable polymer with several advantages over other implant materials approved by the FDA, such as PLGA and PLA. The PHA has a weaker immune response and is suitable for use in medical implants. However, the in vivo biodegradation of PHA is slower than PLA and PLGA, making it unsuitable for rapid in vivo degradation applications [169]. To overcome this constraint and guarantee quick biodegradation, scientists have experimented with mixing PHAs with bigger and smaller molecular sizes. To hasten biodegradation in vitro and in vivo, tests with random or block copolymerization or mixing PHA with PLA or PLGA were successfully performed [169]. Despite these encouraging results, the FDA has exclusively authorized P4HB as a suture material. Many companies are unable to begin the clinical application procedure since it is time-consuming and costly.

The PHA must be purified to remove lipopolysaccharides (when it is produced by gram-negative bacteria), leftover proteins, and other contaminants before it can be used in health applications. To guarantee the delivery of targeted PHA with the necessary constant composition, molecular weights, and purity, this purification method necessitates collaboration with a PHA manufacturer. Property adjustments may be necessary for the required PHA throughout the development phase, and this may only be performed if a partnered producer is willing to provide the necessary PHA material [168]. Despite these difficulties, PHA has many uses in medical implants, including as injectable PHA, artificial blood arteries, heart valves, artificial nerve conduits, lithography, and drug-delivery structures. Among these uses, 3D printing PHA is one that requires careful consideration, particularly when creating unique forms for certain use cases. However, PHA’s sluggish crystallisation could be its biggest deterrent from becoming a viable material for three-dimensional printing. To further this field, scientists are investigating the creation of biocompatible chemicals that may quicken PHA crystallisation. The PHA 3D printing has a large market, particularly in light of the growing need for plastic surgery alongside various medical applications [170].

In conclusion, while PHA offers various environmental and performance advantages, such as biodegradability and customizable properties, its high production cost and processing limitations are important drawbacks for its widespread adoption. Ongoing research might lead to more cost-effective synthesis pathways or ways to streamline the processing procedures. Additionally, emphasizing the long-term environmental benefits, the increasing consumer demand for sustainable alternatives, and the advantages over other biocompatible materials could strengthen the case for wider adoption despite initial challenges [166].

### Current Limitations for PHA Use in Medical and Dental Fields

Due to their hydrophobicity, and brittle mechanical characteristics, the majority of PHA polyesters present limitations for use as biomaterials. For biomedical applications, PHAs should be more amphiphilic than hydrophobic, especially for drug-delivery systems and tissue engineering [171]. Also, the shorter carbon chain lengths of the scl-PHAs and PHBs are shown to result in a greater crystallinity content, which limits their biological uses by making them rigid and inflexible [172].

The crystalline structure of scl-PHAs leads to inadequate mechanical characteristics and less biocompatibility. Copolymerizing PHB with PEG may address these issues by eliminating PHB’s stiffness and brittleness, resulting in a significant enhancement in the Young’s modulus values to 21 MPa. The elongation at break values were greatly improved by up to 1912%. The PHB’s stiffness and hydrophobic nature allow for improvement, whereas PEG’s flexibility and hydrophilic properties contribute to this possibility. By adjusting the length of the PHB and PEG blocks to match the needs of the application, the copolymer’s crystalline structure and water absorption capability may be changed. The biodegradable amphiphilic PHB–PEG polymers shown favourable cell biocompatibility in cytotoxicity assessments [173]. Moreover, enhancing the flexibility of PHB with low crystallinity may be achieved by adding long alkyl side chains like 4HB and 3HV, leading to the formation of copolymers such as P(3HBco4HB) and P(3HBco3HV) [174].

The aforementioned limitations also lead to the emergence of a new category of materials referred to as ‘blends with PHAs’. This emerging material class is being extensively researched to enhance the thermal and mechanical characteristics of PHAs for biological applications [175]. The blend of PHB and PLA is comparatively the most studied blend, with mechanical properties intermediate between the individual components [175].

Zhao et al. (2013) studied the formation of P(3HBco3HV) and PLA blends with improved mechanical properties [176]. According to Zembouai et al. (2013) increasing the amount of PLA in P(3HBco3HV) and PLA blends could enhance their thermal stability. The mcl-PHAs exhibited decreased Tm, Tg, and crystallinity in comparison to scl-PHAs. By extending the length of the pendant chain, the physical properties of mcl-PHAs may be modified to change from semi-crystalline elastomers to amorphous sticky polymeric materials. This modification leads to a decrease in Tg. According to reports, mcl-PHAs with certain side chain lengths are valuable biopolymeric elastomers that have lower tensile strength and higher elongation at break. Increasing the length of the side chain in mcl-PHAs results in the production of sticky biopolymeric materials [174]. The mechanical properties can also be improved when PHB is blended with P(3HBco3HHx). The resulting blend, i.e., P(3HBco3HHx)/PHB blend polymers, showed an improved elongation at break value of 15 to 106% once the content of P(3HBco3HHx) copolymer was increased from 40 to 60% [177].

These restrictions may also be efficiently solved through research including copolymerization with other chemicals. Some of these compounds consist of natural and synthetic rubbers, as well as other polymeric components. For instance, mcl-PHA poly(3-hydroxy undecanoate) (P(3HU)) may be combined with PEG by copolymerization to adjust the mechanical characteristics of the resultant copolymer, known as P(3HU)-co-PEG [178]. By increasing the quantity of PEG in the copolymer, the elongation at break increased by 16% and tensile strength were enhanced to 65 MPa, [179]. Similarly, the biocompatibility of P(3HU)-co-PEG was significantly improved in conjunction with PEG.

The scl-PHAs and mcl-PHAs are valuable polymeric materials that can be processed into elastomers for excellent medical applications [180]. While there have been numerous investigations into the effects of PHAs on cell multiplication and biocompatibility, further evidence is required to confirm the non-carcinogenicity of each cell line further research is required on every cell line to determine the carcinogenicity/non-carcinogenicity impact of PHAs [68].

The biocompatibility of PHAs, including PHB, P(3HBco3HHx), and P(3HBco3HHx)/PHB blends, was evaluated in vitro utilizing the mouse fibroblast cell line L929 (MFCLL929). The L929 cell growth was more efficient on PLA polymers and PHB. Yet, the restriction was resolved by creating composite films of P(3HBco3HHx) and PHB, which promoted the growth of L929 cells. The extent of enhancement varies based on the amount of P(3HBco3HHx) present in the blend films. The surface properties of the P(3HBco3HHx) copolymer were altered as the amount of 3HHx monomeric units increased. The most polished surface was obtained with the copolymer that included 20% HHx monomers. The PHB polymer exhibited the greatest hydrophilicity compared to all the evaluated PHB and co-polymeric P(3HBco3HHx). All copolymeric P(3HBco3HHx) exhibited a strong attraction to protein molecules and had biocompatible characteristics [181].

Additionally, the biocompatibility of these polymeric materials was significantly enhanced by treatment with lipases and sodium hydroxide. It is noteworthy that the biocompatibility of PLA and treated PHB polymers was found to be almost identical, while P(3HB-) copolymers, as well as their dominant blends, exhibited greater biocompatibility than PLA [177].

Wang et al. 2005 examined three-dimensional scaffolds made of PHB and co-polymeric P(3HBco3HHx) utilizing MFCLL929. The surfaces of PHB and co-polymeric P(3HBco3HHx) were more hydrophilic after lipase treatment. L929 cell proliferation on the lipase-treated scaffolds was double that of the control. A 40% decrease in cell proliferation was seen on PHA scaffolds coated with hyaluronan in comparison to the control. Although scaffolds are linked to enhanced hydrophilic characteristics, hyaluronan coating reduced cell adhesion and proliferation on PHAs. Lipase pretreatment enhanced cell growth on PHA scaffolds but did not enhance hydrophilic characteristics compared to hyaluronan coating [181].

Both hydrophilic and hydrophobic properties are essential for the biocompatibility of P(3HBco3HHx), particularly for promoting the growth of L929 cells on its surface.

The PHA polymers, unlike FDA-approved implant substances such as PLA and PLGA, exhibit minimal immunoreactions and slow in vivo biodegradation [141]. Therefore, isolated PHA polymeric materials are unsuitable for applications that require rapid in vivo disintegration. However, combining higher and lower molecular weight PHAs may help maintain desirable mechanical assets while promoting rapid biodegradation [182]. It is thought that random or block co-polymerization, or even mixing PHA with PLA/PLGA, could speed up or improve the rate at which PHA breaks down in the laboratory and in living organisms [183].

The degradation processes of PHB and P(3HBco3HHx) scaffolds may be controlled by altering the ratio of substances with different molecular weights. However, it is regularly observed that the breakdown of these scaffolds occurs at a slower pace compared to the rate of new tissue formation [184]. Therefore, additional research is required to develop PHA polymers that promote tissue regeneration and have a disintegration rate that matches the speed of tissue regeneration.

In addition to these limitations, the production of PHA is hampered by its cost. To reduce expenses, cost-effective and sustainable materials can be used, such as renewable waste materials and by-products from processing industries [163]. These include molasses, palm oil, crude glycerol, waste vegetable oils, agricultural wastes, algae hydrolysates, cheese whey, and wastewater. These materials are available in copious quantities and have a high content of organic matter, making them promising substrates for economical PHA production [57,163,185,186].

Metagenomics and molecular biology advancements may aid in creating improved strains for manufacturing PHA. An optimal industrial strain for PHA synthesis should possess the following traits: non-pathogenic, with a well-defined genomic background, fragile cell wall, simple genetic modification, easy aggregation, rapid growth in a specified medium, absence of toxin production, and potential cellulose utilization. The strains should possess a high capacity for PHA accumulation, be able to grow on mixed substrates, have a high substrate-to-polymer yield, and produce scl and/or mcl PHAs, ideally with a consistent composition. To efficiently extract PHAs, the genetically engineered strain should possess a weak cell wall, high cell size, and easily inducible flocculation [187].

On 27 May 2024, the EU Commission approved its new Green transition, as the Council gave its final approval to the ecodesign regulation that replaces the existing ecodesign directive and enlarges its scope, beyond energy products, to all kind of goods placed in the EU market [188]. Despite the extensive research indicating significant potential for medical applications of PHAs beyond P4HB, their successful commercialization remains contingent upon obtaining regulatory approval from agencies worldwide [31]. Current efforts are concentrated not only on overcoming regulatory challenges but also on generating comprehensive safety and efficacy data mandated by regulatory authorities. Researchers are engaging in active collaboration with these agencies to develop standardized testing protocols, implement quality control measures, and conduct long-term biocompatibility studies. Furthermore, initiatives are underway to expedite the approval process by ensuring that PHAs comply with the rigorous standards set for medical devices and drug-delivery systems. These regulatory improvements, in conjunction with scientific advancements, are crucial for realizing the full potential of PHAs in the biomedical sector.

## 6. Conclusions

Some PHAs, as sustainable, biocompatible, and bioresorbable polymers, have demonstrated their capacity to serve as innovative materials for various medical applications. This family of biopolyesters is being studied for its potential in regenerative medicine and enhanced wound care, in addition to uses in tissue engineering and drug-delivery systems. PHAs with specific properties—like increased stiffness or elasticity—can be designed to satisfy the requirements of many medicinal uses. To further increase the therapeutic potential of PHA-based materials, researchers are also investigating the incorporation of bioactive molecules, such as growth factors or antimicrobial agents. These developments highlight the growing versatility of PHAs as a platform for creating next-generation biomaterials in the medical field.

The research works gathered in this review have underscored the unique properties of some PHAs that make them particularly suitable for dental use. Their ability to degrade in the body without leaving harmful residues aligns with the current demand for materials that can minimize long-term side effects and reduce the need for repeated surgeries. The mechanical strength of some PHA blends and composites, which surpasses that of homopolymers, is especially critical in dental applications where materials are subjected to considerable stress.

Furthermore, the potential of PHAs to sequester pathogenic microorganisms offers a novel approach to preventing and treating oral diseases, with applications in products such as toothpaste, mouthwash, and dental pastes. This antimicrobial property, combined with the ability to tailor PHA formulations for specific dental applications, presents a significant advancement in oral healthcare.

The green perspective of PHAs cannot be overstated. As bioplastics, PHAs represent a shift towards environmentally responsible materials that can be produced at large scale. Their biodegradability and production from renewable resources contribute to a reduced environmental footprint, making them an attractive alternative to traditional, non-degradable dental materials.

PHAs’ widespread use in medical fields is still hampered due to cost, which is higher when compared to other biomaterials for medical application, and to a need for further collaboration among the different contributors in this sector (researchers, health professionals, and regulatory agencies) leading to a more efficient route to their evaluation and approval.

In conclusion, PHAs hold a promising future in dentistry, offering a confluence of biocompatibility, mechanical robustness, and environmental sustainability. As research progresses, it is anticipated that PHAs will play an increasingly vital role in dental material innovation, leading to improved patient outcomes and a greener approach to oral health solutions. The potential of PHAs in dentistry is a testament to their versatility and the broader commitment to sustainable and advanced healthcare practices.

## Figures and Tables

**Table 1:** Melting and glass transition temperature, and mechanical properties of some of the most common scl- and mcl-PHAs—adapted from [21].

PHA	Tm (°C)	Tg (°C)	Tensile Strength (MPa)	Elongation at Break (%)	Young’s Modulus (GPa)
**scl-PHAs**					
P3HB	177–180	4	40–62	5–6	1.1–3.5
P4HB	53–61	−50–−47	13.8–104	696.6–1014	0.149–0.180
P3HV	104–110	−16.5	31.2	14	-
P3HBco4HB	152–164	−45–−7	3–16	268–626	-
P3HBco3HV	133–170	−8–−1	20–38	50	0.2–2.9
**mcl-PHAs**					
PHO	72	−37.2	12	312.9	0.0199
P3HBcoHHx	120–127	−2–−1	9–21	380–850	0.0236

**Table 2:** Summary of PHAs’ main features.

Characteristic	Description	References
Chemical Structure	Polyesters with each repeating unit in the R-configuration Over 150 different monomeric constituents have been reported as microbially synthesized PHAs	[41]
Monomer types	scl-PHAs have ≤5 carbon atoms per monomer (e.g., PHB, P4HB, PHV) mcl-PHAs have 6–14 carbon atoms per monomer (e.g., PHHx, PHO) lcl-PHAs have ≥15 carbon atoms per monomer	[42]
Thermal Properties	Thermoplastics with Tm ranging from ~50 °C to ~180 °C Depending on the type of monomers, they can have a high degree of crystallinity (~80%) or be amorphous materials	[43]
Mechanical Properties	Tensile strength: 15–150 MPa Young’s modulus: 0.21–4 GPa Elongation at break: 1.5–1000% Some are comparable to synthetic plastics like PP, PE, PS	[44]
Biodegradabilty	Biodegradable by microorganisms Degraded in vivo by depolymerases, lipases and esterases, and through surface erosion	[31]
Biocompatibility	scl-PHAs and mcl-PHAs are non-toxic and biocompatible with biological systems when adequately processed 3HB monomers are naturally found in blood of humans and animals	[31]

**Table 3:** PHA in vivo use.

PHA	Applications	References
PHB scaffold with nanobioglass	By considerably increasing osteoblast-like MG63 cell proliferation, bone tissue engineering may lead to improved osteoconductivity	[127]
PHB treated with NaOH	Antibacterial, osteoconductive guided bone regeneration membrane useful in cranio-maxillofacial and dental tissue engineering	[128]
Tricalcium phosphate–PHB composite	Pre-existing cone-beam computed tomography images were utilised to 3D print customised TCP–PHB scaffolds for patients with clefts, according to their unique alveolar bone geometry	[129]

**Table 4:** Recent patents related to the use of PHA in dentistry.

Patent Title	Description	Patent Number	Date of Publication	Reference
Polyhydroxyalkanoate (PHA) for use in the treatment and/or prevention of oral cavity diseases	This patent describes the use of PHA to sequester pathogenic microorganisms in the oral cavity, aiding in disease prevention.	WO2020128584A1	25 June 2020	[156]
Powdered composition based on a polyhydroxyalkanoate and use thereof in dental prophylaxis	This invention involves a powdered PHA composition aimed at dental prophylaxis, highlighting its biodegradability and effectiveness.	EP3813771A1	4 October 2023	[157]
Implantable composites and compositions comprising releasable bioactive agents	This patent is related to composites and compositions that effectively provide a bioactive agent to or near a tissue adjacent to the implantation site that can be applied to a graft or implanted into a subject.	JP6368737B2	1 August 2018	[158]
Aligners having force regeneration	The invention relates to methods and apparatus for the repeated regeneration of strength in materials used for orthodontic aligners.	US20230380938	30 November 2023	[159]
Composite materials for orthodontic applications	Multi-layer composite material assembly made of PHA and other polymers	US20220266577	25 August 2022	[160]
Medical articles with microstructured surface having increased microorganism removal when cleaned and methods	Medical articles with microstructured surface made of PHA	WO2022180466A1	1 September 2022	[161]

## Data Availability

No new data were created or analyzed in this study.

## References

[1] Raza Z.A., Abid S., Banat I.M. (2018). Polyhydroxyalkanoates: Characteristics, production, recent developments and applications. Int. Biodeterior. Biodegrad..

[2] Behera S., Priyadarshanee M., Vandana Das S. (2022). Polyhydroxyalkanoates, the bioplastics of microbial origin: Properties, biochemical synthesis, and their applications. Chemosphere.

[3] He Y., Hu Z., Ren M., Ding C., Chen P., Gu Q., Wu Q. (2014). Evaluation of PHBHHx and PHBV/PLA fibers used as medical sutures. J. Mater. Sci. Mater. Med..

[4] Scholar Google. https://scholar.google.com/.

[5] Scopus. http://www.scopus.com/.

[6] Conte R., De Luise A., Petillo O., Rengo C., Riccitiello F., Di Salle A., Peluso G. (2017). Biodegradable polymers for dental tissue engineering and regeneration. Biodegradable Polymers: Recent Developments and New Perspectives.

[7] Chen T., Pang Z., He S., Li Y., Shrestha S., Little J.M., Yang H., Chung T.C., Sun J., Whitley H.C. (2024). Machine intelligence-accelerated discovery of all-natural plastic substitutes. Nat. Nanotechnol..

[8] Mangal M., Rao C.V., Banerjee T. (2023). Bioplastic: An eco-friendly alternative to non-biodegradable plastic. Polym. Int..

[9] Shahid S., Razzaq S., Farooq R., Nazli Z.I. (2021). Polyhydroxyalkanoates: Next generation natural biomolecules and a solution for the world’s future economy. Int. J. Biol. Macromol..

[10] Lemoigne M. (1926). Products of dehydration and of polymerization of β-hydroxybutyric acid. Bull. Soc. Chem. Biol..

[11] Lee S.Y., Choi J., Wong H.H. (1999). Recent advances in polyhydroxyalkanoate production by bacterial fermentation: Mini-review. Int. J. Biol. Macromol..

[12] Ansari S., Sami N., Yasin D., Ahmad N., Fatma T. (2021). Biomedical applications of environmental friendly poly-hydroxyalkanoates. Int. J. Biol. Macromol..

[13] Peng S.W., Guo X.Y., Shang G.G., Li J., Xu X.Y., You M.L., Li P., Chen G.Q. (2011). An assessment of the risks of carcinogenicity associated with polyhydroxyalkanoates through an analysis of DNA aneuploid and telomerase activity. Biomaterials.

[14] Sharma V., Sehgal R., Gupta R. (2021). Polyhydroxyalkanoate (PHA): Properties and Modifications. Polymer.

[15] Cavalheiro J.M.B.T., Pollet E., Diogo H.P., Cesário M.T., Avérous L., de Almeida M.C.M.D., da Fonseca M.M.R. (2013). On the heterogeneous composition of bacterial polyhydroxyalkanoate terpolymers. Bioresour. Technol..

[16] Vicente D., Proença D.N., Morais P.V. (2023). The Role of Bacterial Polyhydroalkanoate (PHA) in a Sustainable Future: A Review on the Biological Diversity. Int. J. Environ. Res. Public Health.

[17] Singh A.K., Srivastava J.K., Chandel A.K., Sharma L., Mallick N., Singh S.P. (2019). Biomedical applications of microbially engineered polyhydroxyalkanoates: An insight into recent advances, bottlenecks, and solutions. Appl. Microbiol. Biotechnol..

[18] Gao Y., Kong L., Zhang L., Gong Y., Chen G., Zhao N., Zhang X. (2006). Improvement of mechanical properties of poly(dl-lactide) films by blending of poly(3-hydroxybutyrate-co-3-hydroxyhexanoate). Eur. Polym. J..

[19] Martin D.P., Williams S.F. (2003). Medical applications of poly-4-hydroxybutyrate: A strong flexible absorbable biomaterial. Biochem. Eng. J..

[20] Williams S.F., Martin D.P., Moses A.C. (2016). The History of GalaFLEX P4HB Scaffold. Aesthet. Surg. J..

[21] Guo W., Yang K., Qin X., Luo R., Wang H., Huang R. (2022). Polyhydroxyalkanoates in tissue repair and regeneration. Eng. Regen..

[22] Bejagam K.K., Gupta N.S., Lee K.S., Iverson C.N., Marrone B.L., Pilania G. (2022). Predicting the Mechanical Response of Polyhydroxyalkanoate Biopolymers Using Molecular Dynamics Simulations. Polymers.

[23] Boesel L.F., Le Meur S., Thöny-Meyer L., Ren Q. (2014). The effect of molecular weight on the material properties of biosynthesized poly(4-hydroxybutyrate). Int. J. Biol. Macromol..

[24] Tao J., Song C., Cao M., Hu D., Liu L., Liu N., Wang S. (2009). Thermal properties and degradability of poly(propylene carbonate)/poly(β-hydroxybutyrate-co-β-hydroxyvalerate) (PPC/PHBV) blends. Polym. Degrad. Stab..

[25] Chuah J.A., Yamada M., Taguchi S., Sudesh K., Doi Y., Numata K. (2013). Biosynthesis and characterization of polyhydroxyalkanoate containing 5-hydroxyvalerate units: Effects of 5HV units on biodegradability, cytotoxicity, mechanical and thermal properties. Polym. Degrad. Stab..

[26] Gopi S., Kontopoulou M., Ramsay B.A., Ramsay J.A. (2018). Manipulating the structure of medium-chain-length polyhydroxyalkanoate (MCL-PHA) to enhance thermal properties and crystallization kinetics. Int. J. Biol. Macromol..

[27] Grigore M.E., Grigorescu R.M., Iancu L., Ion R.M., Zaharia C., Andrei E.R. (2019). Methods of synthesis, properties and biomedical applications of polyhydroxyalkanoates: A review. J. Biomater. Sci. Polym. Ed..

[28] Nagase K., Shukuwa R., Takahashi H., Takeda N., Okano T. (2020). Enhanced mechanical properties and cell separation with thermal control of PIPAAm-brushed polymer-blend microfibers. J. Mater. Chem. B..

[29] Volova T.G., Shishatskaya E.I., Nikolaeva E.D., Sinskey A.J. (2014). In vivo study of 2D PHA matrices of different chemical compositions: Tissue reactions and biodegradations. Mater. Sci. Technol..

[30] Tang X., Westlie A.H., Caporaso L., Cavallo L., Falivene L., Chen E.Y.-X. (2020). Biodegradable Polyhydroxyalkanoates by Stereoselective Copolymerization of Racemic Diolides: Stereocontrol and Polyolefin-Like Properties. Angew. Chem. Int. Ed..

[31] Pulingam T., Appaturi J.N., Parumasivam T., Ahmad A., Sudesh K. (2022). Biomedical Applications of Polyhydroxyalkanoate in Tissue Engineering. Polymers.

[32] Ross G., Ross S., Tighe B.J. (2016). Bioplastics: New Routes, New Products. Brydson’s Plastics Materials: Eighth Edition.

[33] Volova T., Shishatskaya E., Sevastianov V., Efremov S., Mogilnaya O. (2003). Results of biomedical investigations of PHB and PHB/PHV fibers. Biochem. Eng. J..

[34] Guo K., Martin D.P., Chu C.C. (2015). Poly-4-hydroxybutyrate (P4HB) in Biomedical Applications and Tissue Engineering. Biodegradable Polymers.

[35] Numata K., Abe H., Iwata T. (2009). Biodegradability of Poly(hydroxyalkanoate) Materials. Materials.

[36] Wu C.S. (2014). Mechanical properties, biocompatibility, and biodegradation of cross-linked cellulose acetate-reinforced polyester composites. Carbohydr. Polym..

[37] Egan B., D’Agostino D.P. (2016). Fueling Performance: Ketones Enter the Mix. Cell Metab..

[38] Ang S.L., Shaharuddin B., Chuah J.A., Sudesh K. (2020). Electrospun poly(3-hydroxybutyrate-co-3-hydroxyhexanoate)/silk fibroin film is a promising scaffold for bone tissue engineering. Int. J. Biol. Macromol..

[39] Hu Y.J., Wei X., Zhao W., Liu Y.S., Chen G.Q. (2009). Biocompatibility of poly(3-hydroxybutyrate-co-3-hydroxyvalerate-co-3-hydroxyhexanoate) with bone marrow mesenchymal stem cells. Acta Biomater..

[40] Yan C., Wang Y., Shen X.-Y., Yang G., Jian J., Wang H.-S., Chen G.-Q., Wu Q. (2011). MicroRNA regulation associated chondrogenesis of mouse MSCs grown on polyhydroxyalkanoates. Biomaterials.

[41] Laycock B., Halley P., Pratt S., Werker A., Lant P. (2013). The chemomechanical properties of microbial polyhydroxyalkanoates. Prog. Polym. Sci..

[42] Hahn T., Alzate M.O., Leonhardt S., Tamang P., Zibek S. (2024). Current trends in medium-chain-length polyhydroxyalkanoates: Microbial production, purification, and characterization. Eng. Life Sci..

[43] Mai J., Kockler K., Parisi E., Chan C.M., Pratt S., Laycock B. (2024). Synthesis and physical properties of polyhydroxyalkanoate (PHA)-based block copolymers: A review. Int. J. Biol. Macromol..

[44] Naser A.Z., Deiab I., Darras B.M. (2021). Poly(lactic acid) (PLA) and polyhydroxyalkanoates (PHAs), green alternatives to petroleum-based plastics: A review. RSC Adv..

[45] Philip S., Keshavarz T., Roy I. (2007). Polyhydroxyalkanoates: Biodegradable polymers with a range of applications. J. Chem. Technol. Biotechnol..

[46] Ojumu T.V., Yu J., Solomon B.O. (2004). Production of Polyhydroxyalkanoates, a bacterial biodegradable polymer. Afr. J. Biotechnol..

[47] Cavalheiro J.M.B.T., de Almeida M.C.M.D., Grandfils C., da Fonseca M.M.R. (2009). Poly(3-hydroxybutyrate) production by *Cupriavidus necator* using waste glycerol. Process Biochem..

[48] Yue H., Ling C., Yang T., Chen X., Chen Y., Deng H., Wu Q., Chen J., Chen G.Q. (2014). A seawater-based open and continuous process for polyhydroxyalkanoates production by recombinant *Halomonas campaniensis LS21* grown in mixed substrates. Biotechnol. Biofuels.

[49] Cheng J., Nordeste R., Trainer M.A., Charles T.C. (2017). Methods for the Isolation of Genes Encoding Novel PHA Metabolism Enzymes from Complex Microbial Communities. Methods Mol. Biol..

[50] Saito Y., Nakamura S., Hiramitsu M., Doi Y. (1996). Microbial Synthesis and Properties of Poly(3-hydroxybutyrate-co-4-hydroxybutyrate). Polym. Int..

[51] Kim J.S., Lee B.H., Kim B.S. (2005). Production of poly(3-hydroxybutyrate-co-4-hydroxybutyrate) by *Ralstonia eutropha*. Biochem. Eng. J..

[52] Cesário M.T., Raposo R.S., Almeida MCMD de van Keulen F., Ferreira B.S., Telo J.P., da Fonseca M.M.R. (2014). Production of poly(3-hydroxybutyrate-co-4-hydroxybutyrate) by Burkholderia sacchari using wheat straw hydrolysates and gamma-butyrolactone. Int. J. Biol. Macromol..

[53] Le Meur S., Zinn M., Egli T., Thöny-Meyer L., Ren Q. (2013). Poly(4-hydroxybutyrate) (P4HB) production in recombinant Escherichia coli: P4HB synthesis is uncoupled with cell growth. Microb. Cell Fact..

[54] Borrero-de Acuña J.M., Bielecka A., Häussler S., Schobert M., Jahn M., Wittmann C., Jahn D., Poblete-Castro I. (2014). Production of medium chain length polyhydroxyalkanoate in metabolic flux optimized *Pseudomonas putida*. Microb. Cell Fact..

[55] Vu D.H., Mahboubi A., Root A., Heinmaa I., Taherzadeh M.J., Åkesson D. (2023). Application of Immersed Membrane Bioreactor for Semi-Continuous Production of Polyhydroxyalkanoates from Organic Waste-Based Volatile Fatty Acids. Membranes.

[56] Guimarães T.C., Araújo E.S., Hernández-Macedo M.L., López J.A. (2022). Polyhydroxyalkanoates: Biosynthesis from Alternative Carbon Sources and Analytic Methods: A Short Review. J. Polym. Environ..

[57] Cristea A., Baricz A., Leopold N., Floare C.G., Borodi G., Kacso I., Tripon S., Bulzu P.A., Andrei A.Ș., Cadar O. (2018). Polyhydroxybutyrate production by an extremely halotolerant *Halomonas elongata* strain isolated from the hypersaline meromictic Fără Fund Lake (Transylvanian Basin, Romania). J. Appl. Microbiol..

[58] Mitra R., Xu T., Xiang H., Han J. (2020). Current developments on polyhydroxyalkanoates synthesis by using halophiles as a promising cell factory. Microb. Cell Fact..

[59] Blunt W., Dartiailh C., Sparling R., Gapes D.J., Levin D.B., Cicek N. (2019). Development of High Cell Density Cultivation Strategies for Improved Medium Chain Length Polyhydroxyalkanoate Productivity Using Pseudomonas putida LS46. Bioengineering.

[60] Ramsay J.A., Berger E., Voyer R., Chavarie C., Ramsay B.A. (1994). Extraction of poly-3-hydroxybutyrate using chlorinated solvents. Biotechnol. Tech..

[61] Kurian N.S., Das B. (2021). Comparative analysis of various extraction processes based on economy, eco-friendly, purity and recovery of polyhydroxyalkanoate: A review. Int. J. Biol. Macromol..

[62] Jacquel N., Lo C.W., Wei Y.H., Wu H.S., Wang S.S. (2008). Isolation and purification of bacterial poly(3-hydroxyalkanoates). Biochem. Eng. J..

[63] Pagliano G., Galletti P., Samorì C., Zaghini A., Torri C. (2021). Recovery of Polyhydroxyalkanoates from Single and Mixed Microbial Cultures: A Review. Front. Bioeng. Biotechnol..

[64] Rosengart A., Cesário M.T., de Almeida M.C.M.D., Raposo R.S., Espert A., de Apodaca E.D., da Fonseca M.M.R. (2015). Efficient PHB extraction from *Burkholderia sacchari* cells using non-chlorinated solvents. Biochem. Eng. J..

[65] Shi F., Ashby R.D., Gross R.A. (1997). Fractionation and Characterization of Microbial Polyesters Containing 3-Hydroxybutyrate and 4-Hydroxybutyrate Repeat Units. Macromolecules.

[66] Feres M., Figueiredo L.C., Soares G.M.S., Faveri M. (2015). Systemic antibiotics in the treatment of periodontitis. Periodontol. 2000.

[67] Frederick S. (2008). Biocompatibility of Materials in Medical Devices. Wiley Encyclopedia of Chemical Biology.

[68] Ali I., Jamil N. (2016). Polyhydroxyalkanoates: Current applications in the medical field. Front. Biol..

[69] Vastano M., Casillo A., Corsaro M.M., Sannia G., Pezzella C. (2015). Production of medium chain length polyhydroxyalkanoates from waste oils by recombinant *Escherichia coli*. Eng. Life Sci..

[70] Urbina L., Wongsirichot P., Corcuera M.Á., Gabilondo N., Eceiza A., Winterburn J., Retegi A. (2018). Application of cider by-products for medium chain length polyhydroxyalkanoate production by Pseudomonas putida KT2440. Eur. Polym. J..

[71] Butt F.I., Muhammad N., Hamid A., Moniruzzaman M., Sharif F. (2018). Recent progress in the utilization of biosynthesized polyhydroxyalkanoates for biomedical applications–Review. Int. J. Biol. Macromol..

[72] Sahana T.G., Rekha P.D. (2018). Biopolymers: Applications in wound healing and skin tissue engineering. Mol. Biol. Rep..

[73] Marcello E., Maqbool M., Nigmatullin R., Cresswell M., Jackson P.R., Basnett P., Knowles J.C., Boccaccini A.R., Roy I. (2021). Antibacterial Composite Materials Based on the Combination of Polyhydroxyalkanoates with Selenium and Strontium Co-substituted Hydroxyapatite for Bone Regeneration. Front. Bioeng. Biotechnol..

[74] Basnett P., Matharu R.K., Taylor C.S., Illangakoon U., Dawson J.I., Kanczler J.M., Behbehani M., Humphrey E., Majid Q., Lukasiewicz B. (2021). Harnessing Polyhydroxyalkanoates and Pressurized Gyration for Hard and Soft Tissue Engineering. ACS Appl. Mater. Interfaces.

[75] Mai R., Hagedorn M.G., Gelinsky M., Werner C., Turhani D., Spath H., Gedrange T., Lauer G. (2006). Ectopic bone formation in nude rats using human osteoblasts seeded poly(3)hydroxybutyrate embroidery and hydroxyapatite-collagen tapes constructs. J. Cranio-Maxillofac. Surg..

[76] Mačković M., Hoppe A., Detsch R., Mohn D., Stark W.J., Spiecker E., Boccaccini A.R. (2012). Bioactive glass (type 45S5) nanoparticles: In vitro reactivity on nanoscale and biocompatibility. J. Nanopart. Res..

[77] Shuai C., Guo W., Gao C., Yang Y., Xu Y., Liu L., Qin T., Sun H., Yang S., Feng P. (2017). Calcium Silicate Improved Bioactivity and Mechanical Properties of Poly(3-hydroxybutyrate-co-3-hydroxyvalerate) Scaffolds. Polymers.

[78] Amini A.R., Laurencin C.T., Nukavarapu S.P. (2012). Bone Tissue Engineering: Recent Advances and Challenges. Crit. Rev. Biomed. Eng..

[79] Volkov A.V., Muraev A.A., Zharkova I.I., Voinova V.V., Akoulina E.A., Zhuikov V.A., Khaydapova D.D., Chesnokova D.V., Menshikh K.A., Dudun A.A. (2020). Poly(3-hydroxybutyrate)/hydroxyapatite/alginate scaffolds seeded with mesenchymal stem cells enhance the regeneration of critical-sized bone defect. Mater. Sci. Eng. C.

[80] Stevens M.M. (2008). Biomaterials for bone tissue engineering. Mater. Today.

[81] Zhong L., Hu D., Qu Y., Peng J., Huang K., Lei M., Wu T., Xiao Y., Gu Y., Qian Z. (2019). Preparation of Adenosine-Loaded Electrospun Nanofibers and Their Application in Bone Regeneration. J. Biomed. Nanotechnol..

[82] Cool S.M., Kenny B., Wu A., Nurcombe V., Trau M., Cassady A.I., Grøndahl L. (2007). Poly(3-hydroxybutyrate-*co*-3-hydroxyvalerate) composite biomaterials for bone tissue regeneration: In vitro performance assessed by osteoblast proliferation, osteoclast adhesion and resorption, and macrophage proinflammatory response. J. Biomed. Mater. Res. A.

[83] Chernozem R.V., Surmeneva M.A., Shkarina S.N., Loza K., Epple M., Ulbricht M., Cecilia A., Krause B., Baumbach T., Abalymov A.A. (2019). Piezoelectric 3-D Fibrous Poly(3-hydroxybutyrate)-Based Scaffolds Ultrasound-Mineralized with Calcium Carbonate for Bone Tissue Engineering: Inorganic Phase Formation, Osteoblast Cell Adhesion, and Proliferation. ACS Appl. Mater. Interfaces.

[84] George A., Varghese H., Chandran A., Surendran K.P., Gowd E.B. (2024). Directional freezing-induced self-poled piezoelectric nylon 11 aerogels as high-performance mechanical energy harvesters. J. Mater. Chem. A Mater..

[85] Chen G.Q., Wu Q. (2005). The application of polyhydroxyalkanoates as tissue engineering materials. Biomaterials.

[86] Chen Z., Fu F., Yu Y., Wang H., Shang Y., Zhao Y. (2019). Cardiomyocytes-Actuated Morpho Butterfly Wings. Adv. Mater..

[87] Sodian R., Hoerstrup S.P., Sperling J.S., Daebritz S.H., Martin D.P., Schoen F.J., Vacanti J.P., Mayer J.E. (2000). Tissue engineering of heart valves: In vitro experiences. Ann. Thorac. Surg..

[88] Wu S., Liu Y., Cui B., Qu X., Chen G. (2007). Study on Decellularized Porcine Aortic Valve/Poly (3-hydroxybutyrate-co-3-hydroxyhexanoate) Hybrid Heart Valve in Sheep Model. Artif. Organs.

[89] Motta S.E., Lintas V., Fioretta E.S., Dijkman P.E., Putti M., Caliskan E., Rodriguez Cetina Biefer H., Lipiski M., Sauer M. (2019). Human cell-derived tissue-engineered heart valve with integrated Valsalva sinuses: Towards native-like transcatheter pulmonary valve replacements. npj Regen. Med..

[90] Perry T.E., Kaushal S., Sutherland F.W.H., Guleserian K.J., Bischoff J., Sacks M., Mayer J.E. (2003). Bone marrow as a cell source for tissue engineering heart valves. Ann. Thorac. Surg..

[91] Bagdadi A.V., Safari M., Dubey P., Basnett P., Sofokleous P., Humphrey E., Locke I., Edirisinghe M., Terracciano C., Boccaccini A.R. (2018). Poly(3-hydroxyoctanoate), a promising new material for cardiac tissue engineering. J. Tissue Eng. Regen. Med..

[92] Castellano D., Blanes M., Marco B., Cerrada I., Ruiz-Saurí A., Pelacho B., Araña M., Montero J.A., Cambra V., Prosper F. (2014). A Comparison of Electrospun Polymers Reveals Poly(3-Hydroxybutyrate) Fiber as a Superior Scaffold for Cardiac Repair. Stem Cells Dev..

[93] Mohanna P.-N., Young R.C., Wiberg M., Terenghi G. (2003). A composite poly-hydroxybutyrate–glial growth factor conduit for long nerve gap repairs. J. Anat..

[94] Lizarraga-Valderrama L.R., Panchal B., Thomas C., Boccaccini A.R., Roy I. (2016). Biomedical Applications of Polyhydroxyalkanoates. Biomaterials from Nature for Advanced Devices and Therapies.

[95] Bian Y.Z., Wang Y., Aibaidoula G., Chen G.Q., Wu Q. (2009). Evaluation of poly(3-hydroxybutyrate-co-3-hydroxyhexanoate) conduits for peripheral nerve regeneration. Biomaterials.

[96] Yucel D., Kose G.T., Hasirci V. (2010). Polyester based nerve guidance conduit design. Biomaterials.

[97] Prabhakaran M.P., Vatankhah E., Ramakrishna S. (2013). Electrospun aligned PHBV/collagen nanofibers as substrates for nerve tissue engineering. Biotechnol. Bioeng..

[98] Masaeli E., Morshed M., Nasr-Esfahani M.H., Sadri S., Hilderink J., van Apeldoorn A., van Blitterswijk C.A., Moroni L. (2013). Fabrication, Characterization and Cellular Compatibility of Poly(Hydroxy Alkanoate) Composite Nanofibrous Scaffolds for Nerve Tissue Engineering. PLoS ONE.

[99] Saniye S., Melis K. (2018). Biosensing Devices: Conjugated Polymer Based Scaffolds. Encyclopedia of Polymer Applications.

[100] Manavitehrani I., Fathi A., Badr H., Daly S., Negahi Shirazi A., Dehghani F. (2016). Biomedical Applications of Biodegradable Polyesters. Polymers.

[101] Chu C.C. (2013). Materials for absorbable and nonabsorbable surgical sutures. Biotextiles as Medical Implants.

[102] Odermatt E.K., Funk L., Bargon R., Martin D.P., Rizk S., Williams S.F. (2012). MonoMax Suture: A New Long-Term Absorbable Monofilament Suture Made from Poly-4-Hydroxybutyrate. Int. J. Polym. Sci..

[103] Dydak K., Junka A., Nowacki G., Paleczny J., Szymczyk-Ziółkowska P., Górzyńska A., Aniołek O., Bartoszewicz M. (2022). In Vitro Cytotoxicity, Colonisation by Fibroblasts and Antimicrobial Properties of Surgical Meshes Coated with Bacterial Cellulose. Int. J. Mol. Sci..

[104] Kischkel S., Grabow N., Püschel A., Erdle B., Kabelitz M., Martin D.P., Williams S.F., Bombor I., Sternberg K., Schmitz K.P. (2016). Biodegradable polymeric stents for vascular application in a porcine carotid artery model. Gefässchirurgie.

[105] Panaitescu D.M., Lupescu I., Frone A.N., Chiulan I., Nicolae C.A., Tofan V., Stefaniu A., Somoghi R., Trusca R. (2017). Medium Chain-Length Polyhydroxyalkanoate Copolymer Modified by Bacterial Cellulose for Medical Devices. Biomacromolecules.

[106] Dabiri G., Damstetter E., Phillips T. (2016). Choosing a Wound Dressing Based on Common Wound Characteristics. Adv. Wound Care.

[107] Shishatskaya E.I., Volova T.G. (2004). A comparative investigation of biodegradable polyhydroxyalkanoate films as matrices for in vitro cell cultures. J. Mater. Sci. Mater. Med..

[108] Vigneswari S., Gurusamy T.P., Khairul W.M., Abdul Khalil H.P.S., Ramakrishna S., Amirul A.-A.A. (2021). Surface Characterization and Physiochemical Evaluation of P(3HB-*co*-4HB)-Collagen Peptide Scaffolds with Silver Sulfadiazine as Antimicrobial Agent for Potential Infection-Resistance Biomaterial. Polymers.

[109] Vigneswari S., Murugaiyah V., Kaur G., Abdul Khalil H.P.S., Amirul A.A. (2016). Simultaneous dual syringe electrospinning system using benign solvent to fabricate nanofibrous P(3HB-co-4HB)/collagen peptides construct as potential leave-on wound dressing. Mater. Sci. Eng. C.

[110] Galateasurgical. www.galateasurgical.com.

[111] Pooja B. (2014). Biosynthesis of Polyhydroxyalkanoates, Their Novel Blends and Composites for Biomedical Applications. Ph.D. Thesis.

[112] Shrivastav A., Kim H.Y., Kim Y.R. (2013). Advances in the Applications of Polyhydroxyalkanoate Nanoparticles for Novel Drug Delivery System. BioMed Res. Int..

[113] Fenton O.S., Olafson K.N., Pillai P.S., Mitchell M.J., Langer R. (2018). Advances in Biomaterials for Drug Delivery. Adv. Mater..

[114] Elmowafy E., Abdal-Hay A., Skouras A., Tiboni M., Casettari L., Guarino V. (2019). Polyhydroxyalkanoate (PHA): Applications in drug delivery and tissue engineering. Expert Rev. Med. Devices.

[115] Pecorini G., Ferraro E., Puppi D. (2023). Polymeric Systems for the Controlled Release of Flavonoids. Pharmaceutics.

[116] Adepu S., Ramakrishna S. (2021). Controlled Drug Delivery Systems: Current Status and Future Directions. Molecules.

[117] Iqbal H.M.N., Keshavarz T. (2018). Bioinspired polymeric carriers for drug delivery applications. Stimuli Responsive Polymeric Nanocarriers for Drug Delivery Applications, Volume 1.

[118] Rai R., Keshavarz T., Roether J.A., Boccaccini A.R., Roy I. (2011). Medium chain length polyhydroxyalkanoates, promising new biomedical materials for the future. Mater. Sci. Eng. R Rep..

[119] Pramual S., Assavanig A., Bergkvist M., Batt C.A., Sunintaboon P., Lirdprapamongkol K., Svasti J., Niamsiri N. (2016). Development and characterization of bio-derived polyhydroxyalkanoate nanoparticles as a delivery system for hydrophobic photodynamic therapy agents. J. Mater. Sci. Mater. Med..

[120] Susan M., Baldea I., Senila S., Macovei V., Dreve S., Ion R.M., Cosgarea R. (2011). Photodamaging effects of porphyrins and chitosan on primary human keratinocytes and carcinoma cell cultures. Int. J. Dermatol..

[121] Neagu M., Manda G., Constantin C., Radu E., Ion R.M. (2007). Synthetic porphyrins in experimental photodynamic therapy induce a different antitumoral effect. J. Porphyr. Phthalocyanines.

[122] Kilicay E., Erdal E., Elci P., Hazer B., Denkbas E.B. (2024). Tumour-specific hybrid nanoparticles in therapy of breast cancer. J. Microencapsul..

[123] Babos G., Rydz J., Kawalec M., Klim M., Fodor-Kardos A., Trif L., Feczkó T. (2020). Poly(3-Hydroxybutyrate)-Based Nanoparticles for Sorafenib and Doxorubicin Anticancer Drug Delivery. Int. J. Mol. Sci..

[124] Murueva A.V., Dudaev A.E., Shishatskaya E.I., Ghorabe F.D.E., Nemtsev I.V., Lukyanenko A.V., Volova T.G. (2024). Biodegradable polymer casting films for drug delivery and cell culture. Giant.

[125] Ding Y.W., Li Y., Zhang Z.W., Dao J.W., Wei D.X. (2024). Hydrogel forming microneedles loaded with VEGF and Ritlecitinib/polyhydroxyalkanoates nanoparticles for mini-invasive androgenetic alopecia treatment. Bioact. Mater..

[126] Kalia V.C., Patel S.K.S., Lee J.K. (2023). Exploiting Polyhydroxyalkanoates for Biomedical Applications. Polymers.

[127] Hajiali H., Hosseinalipour M., Karbasi S., Shokrgozar M.A. (2012). The influence of bioglass nanoparticles on the biodegradation and biocompatibility of poly (3-hydroxybutyrate) scaffolds. Int. J. Artif. Organs.

[128] Karahaliloğlu Z., Ercan B., Taylor E.N., Chung S., Denkbaş E.B., Webster T.J. (2015). Antibacterial Nanostructured Polyhydroxybutyrate Membranes for Guided Bone Regeneration. J. Biomed. Nanotechnol..

[129] Berger M., Probst F., Schwartz C., Cornelsen M., Seitz H., Ehrenfeld M., Otto S. (2015). A concept for scaffold-based tissue engineering in alveolar cleft osteoplasty. J. Craniomaxillofac. Surg..

[130] Dwivedi R., Pandey R., Kumar S., Mehrotra D. (2020). Polyhydroxyalkanoates (PHA): Role in bone scaffolds. J. Oral. Biol. Craniofac. Res..

[131] Mi C.H., Qi X.Y., Ding Y.W., Zhou J., Dao J.W., Wei D.X. (2023). Recent advances of medical polyhydroxyalkanoates in musculoskeletal system. Biomater. Transl..

[132] Puppi D., Morelli A., Chiellini F. (2017). Additive Manufacturing of Poly(3-hydroxybutyrate-co-3-hydroxyhexanoate)/poly(ε-caprolactone) Blend Scaffolds for Tissue Engineering. Bioengineering.

[133] Wang M., Duan B. (2011). Nanocomposite Scaffolds for Bone Tissue Engineering: Design, Fabrication, Surface Modification and Sustained Release of Growth Factor. MRS Proc..

[134] Neves N.M., Reis R.L. (2016). Biomaterials from Nature for Advanced Devices and Therapies.

[135] Doineau E., Perdrier C., Allayaud F., Blanchet E., Preziosi-Belloy L., Grousseau E., Gontard N., Angellier-Coussy H. (2023). Designing poly(3-hydroxybutyrate-co-3-hydroxyvalerate) P(3HB-co-3HV) films with tailored mechanical properties. Mater. Today Commun..

[136] Vilar G., Tulla-Puche J., Albericio F. (2012). Polymers and Drug Delivery Systems. Curr. Drug Deliv..

[137] Phuegyod S., Pramual S., Wattanavichean N., Assawajaruwan S., Amornsakchai T., Sukho P., Svasti J., Surarit R., Niamsiri N. (2023). Microbial Poly(hydroxybutyrate-co-hydroxyvalerate) Scaffold for Periodontal Tissue Engineering. Polymers.

[138] Hollister S.J. (2005). Porous scaffold design for tissue engineering. Nat. Mater..

[139] Loh Q.L., Choong C. (2013). Three-Dimensional Scaffolds for Tissue Engineering Applications: Role of Porosity and Pore Size. Tissue Eng. Part B Rev..

[140] Galgut P., Pitrola R., Waite I., Doyle C., Smith R. (1991). Histological evaluation of biodegradable and non-degradable membranes placed transcutaneously in rats. J. Clin. Periodontol..

[141] Qu X., Wu Q., Zhang K., Chen G. (2006). In vivo studies of poly(3-hydroxybutyrate-co-3-hydroxyhexanoate) based polymers: Biodegradation and tissue reactions. Biomaterials.

[142] Fitzpatrick S.G., Cohen D.M., Clark A.N. (2019). Ulcerated Lesions of the Oral Mucosa: Clinical and Histologic Review. Head Neck Pathol..

[143] Owji N., Mandakhbayar N., Gregory D.A., Marcello E., Kim H.W., Roy I., Knowles J.C. (2021). Mussel Inspired Chemistry and Bacteria Derived Polymers for Oral Mucosal Adhesion and Drug Delivery. Front. Bioeng. Biotechnol..

[144] Chen X., Wu G., Feng Z., Dong Y., Zhou W., Li B., Bai S., Zhao Y. (2016). Advanced biomaterials and their potential applications in the treatment of periodontal disease. Crit. Rev. Biotechnol..

[145] Kilicarslan M., Koerber M., Bodmeier R. (2014). In situ forming implants for the delivery of metronidazole to periodontal pockets: Formulation and drug release studies. Drug Dev. Ind. Pharm..

[146] Khodir W.W.A., Guarino V., Alvarez-Perez M., Cafiero C., Ambrosio L. (2013). Trapping tetracycline-loaded nanoparticles into polycaprolactone fiber networks for periodontal regeneration therapy. J. Bioact. Compat. Polym..

[147] Srirangarajan S., Mundargi R.C., Ravindra S., Setty S.B., Aminabhavi T.M., Thakur S. (2011). Randomized, Controlled, Single-Masked, Clinical Study to Compare and Evaluate the Efficacy of Microspheres and Gel in Periodontal Pocket Therapy. J. Periodontol..

[148] Schou S. (2008). Implant treatment in periodontitis-susceptible patients: A systematic review. J. Oral Rehabil..

[149] Weiser J.R., Saltzman W.M. (2014). Controlled release for local delivery of drugs: Barriers and models. J. Control. Release.

[150] Panith N., Assavanig A., Lertsiri S., Bergkvist M., Surarit R., Niamsiri N. (2016). Development of tunable biodegradable polyhydroxyalkanoates microspheres for controlled delivery of tetracycline for treating periodontal disease. J. Appl. Polym. Sci..

[151] Coated VICRYL RAPIDETM (Polyglactin 910) Suture Synthetic Absorbable Suture, Undyed Braided Non-U.S.P. http://synthes.vo.llnwd.net/o16/LLNWMB8/IFUs/JJMDC/ETHICON/LAB-0014998v1%20-%20Vicryl%20Rapide.pdf.

[152] Gregory D.A., Taylor C.S., Fricker A.T.R., Asare E., Tetali S.S.V., Haycock J.W., Roy I. (2022). Polyhydroxyalkanoates and their advances for biomedical applications. Trends Mol. Med..

[153] Panchal M., Khare S., Khamkar P., Suresh Bhole K. (2022). Dental implants: A review of types, design analysis, materials, additive manufacturing methods, and future scope. Mater. Today Proc..

[154] Chen P., Nikoyan L. (2021). Guided Implant Surgery. Dent. Clin. N. Am..

[155] Gielisch M., Heimes D., Thiem D.G.E., Boesing C., Krumpholtz M., Al-Nawas B., Kämmerer P.W. (2022). Steam-sterilized and degradable fused filament fabrication-printed polylactide/polyhydroxyalkanoate surgical guides for dental implants: Are they accurate enough for static navigation?. Int. J. Bioprint..

[156] Saettone P., Condo C., Cellante L., Cicognani V., Alifano P., Calcagnile M., Franchini M.C. (2020). Polyhydroxyalkanoate (pha) for Use in the Treatment and/or Prevention of Oral Cavity Diseases. Italy Patent.

[157] Ferraro A., Saettone P., Franchini M.C. (2023). Powdered Composition Based on a Polyhydroxyalkanoate and Use Thereof in Dental Prophylaxis. Italy Patent.

[158] Tiptom A.J., Burton K.W., Toce T.R., Bowman H., Biggs D., Markland P. (2018). Implantable Composites and Compositions Comprising Releasable Bioactive Agents. International Patent.

[159] Krishnamohan S., Huafeng W. (2023). Aligners Having Force Regeneration. U.S. Patent.

[160] Krishnamohan S., Huafeng W. (2022). Composite Materials for Orthodontic Applications. U.S. Patent.

[161] Jodi L., Connell Raymond P. (2022). Medical Articles with Microstructured Surface Having Increased Microorganism Removal When Cleaned and Methods Thereof. International Patent.

[162] Shao P., Niu B., Chen H., Sun P. (2018). Fabrication and characterization of tea polyphenols loaded pullulan-CMC electrospun nanofiber for fruit preservation. Int. J. Biol. Macromol..

[163] Zytner P., Kumar D., Elsayed A., Mohanty A., Ramarao B.V., Misra M. (2023). A review on polyhydroxyalkanoate (PHA) production through the use of lignocellulosic biomass. RSC Sustain..

[164] Kim D.Y., Kim Y.B., Rhee Y.H. (2000). Evaluation of various carbon substrates for the biosynthesis of polyhydroxyalkanoates bearing functional groups by Pseudomonas putida. Int. J. Biol. Macromol..

[165] Raposo R.S., de Almeida M.C.M.D., da Fonseca M.M.R., Cesário M.T. (2017). Feeding strategies for tuning poly (3-hydroxybutyrate-co-4-hydroxybutyrate) monomeric composition and productivity using Burkholderia sacchari. Int. J. Biol. Macromol..

[166] Alavi S., Thomas S., Sandeep K.P., Kalarikkal N., Varghese J., Yaragalla S. (2014). Polymers for Packaging Applications.

[167] Kumar M., Gupta A., Thakur I.S. (2016). Carbon dioxide sequestration by chemolithotrophic oleaginous bacteria for production and optimization of polyhydroxyalkanoate. Bioresour. Technol..

[168] Ma Y.M., Wei D.X., Yao H., Wu L.P., Chen G.Q. (2016). Synthesis, Characterization and Application of Thermoresponsive Polyhydroxyalkanoate-*graft*-Poly(*N*-isopropylacrylamide). Biomacromolecules.

[169] Li Z., Lim J. (2018). Biodegradable polyhydroxyalkanoates nanocarriers for drug delivery applications. Stimuli Responsive Polymeric Nanocarriers for Drug Delivery Applications, Volume 1.

[170] Chen G.Q., Hajnal I. (2015). The ‘PHAome’. Trends Biotechnol..

[171] Yang X., Zhao K., Chen G.Q. (2002). Effect of surface treatment on the biocompatibility of microbial polyhydroxyalkanoates. Biomaterials.

[172] Brzeska J., Heimowska A., Janeczek H., Kowalczuk M., Rutkowska M. (2014). Polyurethanes Based on Atactic Poly[(R,S)-3-hydroxybutyrate]: Preliminary Degradation Studies in Simulated Body Fluids. J. Polym. Environ..

[173] Loh X.J., Wang X., Li H., Li X., Li J. (2007). Compositional study and cytotoxicity of biodegradable poly(ester urethane)s consisting of poly[(R)-3-hydroxybutyrate] and poly(ethylene glycol). Mater. Sci. Eng. C.

[174] Zembouai I., Kaci M., Bruzaud S., Benhamida A., Corre Y.M., Grohens Y. (2013). A study of morphological, thermal, rheological and barrier properties of Poly(3-hydroxybutyrate-Co-3-Hydroxyvalerate)/polylactide blends prepared by melt mixing. Polym. Test..

[175] Visakh P.M. (2014). Polyhydroxyalkanoates (PHAs), their Blends, Composites and Nanocomposites: State of the Art, New Challenges and Opportunities. Polyhydroxyalkanoate (PHA) Based Blends, Composites and Nanocomposites.

[176] Zhao H., Cui Z., Sun X., Turng L.S., Peng X. (2013). Morphology and Properties of Injection Molded Solid and Microcellular Polylactic Acid/Polyhydroxybutyrate-Valerate (PLA/PHBV) Blends. Ind. Eng. Chem. Res..

[177] Zhao J. (2003). Dual Subcellular Distribution of Cytochrome b5 in Plant, Cauliflower, Cells. J. Biochem..

[178] Chung C.W., Kim H.W., Kim Y.B., Rhee Y.H. (2003). Poly(ethylene glycol)-grafted poly(3-hydroxyundecenoate) networks for enhanced blood compatibility. Int. J. Biol. Macromol..

[179] Ghalia M.A., Dahman Y. (2017). Investigating the effect of multi-functional chain extenders on PLA/PEG copolymer properties. Int. J. Biol. Macromol..

[180] Ye H., Zhang K., Kai D., Li Z., Loh X.J. (2018). Polyester elastomers for soft tissue engineering. Chem. Soc. Rev..

[181] Wang Y.W., Yang F., Wu Q., Cheng Y.Y., Yu P.H.F., Chen J., Chen G.Q. (2005). Effect of composition of poly(3-hydroxybutyrate-co-3-hydroxyhexanoate) on growth of fibroblast and osteoblast. Biomaterials.

[182] Shangguan Y.Y., Wang Y.W., Wu Q., Chen G.Q. (2006). The mechanical properties and in vitro biodegradation and biocompatibility of UV-treated poly(3-hydroxybutyrate-co-3-hydroxyhexanoate). Biomaterials.

[183] Zhang J., Shishatskaya E.I., Volova T.G., da Silva L.F., Chen G.Q. (2018). Polyhydroxyalkanoates (PHA) for therapeutic applications. Mater. Sci. Eng. C.

[184] Young R.C., Terenghi G., Wiberg M. (2002). Poly-3-hydroxybutyrate (PHB): A resorbable conduit for long-gap repair in peripheral nerves. Br. J. Plast. Surg..

[185] Costa S.S., Miranda A.L., de Morais M.G., Costa J.A.V., Druzian J.I. (2019). Microalgae as source of polyhydroxyalkanoates (PHAs)—A review. Int. J. Biol. Macromol..

[186] Roja K., Ruben Sudhakar D., Anto S., Mathimani T. (2019). Extraction and characterization of polyhydroxyalkanoates from marine green alga and cyanobacteria. Biocatal. Agric. Biotechnol..

[187] Khamkong T., Penkhrue W., Lumyong S. (2022). Optimization of Production of Polyhydroxyalkanoates (PHAs) from Newly Isolated Ensifer sp. Strain HD34 by Response Surface Methodology. Processes.

[188] Green Transition: Council Gives Its Final Approval to the Ecodesign Regulation. https://www.consilium.europa.eu/en/press/press-releases/2024/05/27/green-transition-council-gives-its-final-approval-to-the-ecodesign-regulation/.

